# Fully automated point-of-care differential diagnosis of acute febrile illness

**DOI:** 10.1371/journal.pntd.0009177

**Published:** 2021-02-25

**Authors:** Sebastian Hin, Benjamin Lopez-Jimena, Mohammed Bakheit, Vanessa Klein, Seamus Stack, Cheikh Fall, Amadou Sall, Khalid Enan, Mohamed Mustafa, Liz Gillies, Viorel Rusu, Sven Goethel, Nils Paust, Roland Zengerle, Sieghard Frischmann, Manfred Weidmann, Konstantinos Mitsakakis

**Affiliations:** 1 Laboratory for MEMS Applications, IMTEK – Department of Microsystems Engineering, University of Freiburg, Freiburg, Germany; 2 Institute of Aquaculture, University of Stirling, Scotland, United Kingdom; 3 Mast Group Ltd, Mast House, Bootle, Liverpool, United Kingdom; 4 Mast Diagnostica GmbH, Reinfeld, Germany; 5 Arbovirus and viral haemorrhagic fever unit, Institut Pasteur de Dakar, Dakar, Senegal; 6 Department of Virology, Central Laboratory-The Ministry of Higher Education and Scientific Research, Khartoum, Sudan; 7 MagnaMedics Diagnostics BV, Geleen, The Netherlands; 8 Hahn-Schickard, Freiburg, Germany; International Vaccine Institute, REPUBLIC OF KOREA

## Abstract

**Background:**

In this work, a platform was developed and tested to allow to detect a variety of candidate viral, bacterial and parasitic pathogens, for acute fever of unknown origin. The platform is based on a centrifugal microfluidic cartridge, the LabDisk (“FeverDisk” for the specific application), which integrates all necessary reagents for sample-to-answer analysis and is processed by a compact, point-of-care compatible device.

**Methodology/Principal findings:**

A sample volume of 200 μL per FeverDisk was used. *In situ* extraction with pre-stored reagents was achieved by bind-wash-elute chemistry and magnetic particles. Enzymes for the loop-mediated isothermal amplification (LAMP) were pre-stored in lyopellet form providing stability and independence from the cold chain. The total time to result from sample inlet to read out was 2 h. The proof-of-principle was demonstrated in three small-scale feasibility studies: in Dakar, Senegal and Khartoum, Sudan we tested biobanked samples using 29 and 9 disks, respectively; in Reinfeld, Germany we tested spiked samples and analyzed the limit of detection using three bacteria simultaneously spiked in whole blood using 15 disks. Overall during the three studies, the FeverDisk detected dengue virus (different serotypes), chikungunya virus, *Plasmodium falciparum*, *Salmonella enterica* Typhi, *Salmonella enterica* Paratyphi A and *Streptococcus pneumoniae*.

**Conclusions/Significance:**

The FeverDisk proved to be universally applicable as it successfully detected all different types of pathogens as single or co-infections, while it also managed to define the serotype of un-serotyped dengue samples. Thirty-eight FeverDisks at the two African sites provided 59 assay results, out of which 51 (86.4%) were confirmed with reference assay results. The results provide a promising outlook for future implementation of the platform in larger prospective clinical studies for defining its clinical sensitivity and specificity. The technology aims to provide multi-target diagnosis of the origins of fever, which will help fight lethal diseases and the incessant rise of antimicrobial resistance.

## Author summary

Infectious diseases in tropical regions may have a variety of viral, bacterial or parasitic origins and a patient may suffer from several diseases simultaneously, each presenting with acute fever as a clinical symptom. This makes it difficult to determine the origin of the pathogen causing the disease(s). In addition to the endemic infectious diseases, outbreaks of epidemics frequently complicate diagnostic demands. Accurate diagnosis for proper patient management requires the utilization of highly sensitive and specific, rapid, easy-to-use diagnostic tools compatible with point-of-care settings. We describe the use of a disk-shaped microfluidic platform, the “FeverDisk”, for differential diagnosis of acute fever. Our FeverDisk platform demonstrated its capability to detect bacteria, viruses and parasites that are typical of tropical single and co-infections from biobanked samples within only 2 hours and in very good agreement with reference method results. This, in combination with its easy-to-use and point-of-care compatible nature, render our platform a promising candidate for detection of tropical diseases and precise identification of the cause of acute fever, in endemic and epidemic settings. Future work will involve extensive clinical characterization of the platform in prospective studies.

## Introduction

Acute fever is a common symptom observed in patients suffering from infectious diseases [1]. There is a broad range of infections that may cause acute fever. Some of them are accompanied by localized symptoms, e.g. respiratory tract, urinary tract, gastrointestinal infections, while others not, in which case it is termed as non-specific fever, or fever of unknown origin. The latter group of infections frequently occurs in tropical areas. A main cause (etiology) of non-specific fever is *Plasmodium* spp, which, despite the intense efforts and remarkable progress for its eradication, was responsible for 219 million new cases and 435,000 deaths reported globally in 2017, the vast majority of which (90%) were reported in the Sub-Saharan African region [2]. Recent studies investigated the etiologies of fever and found a broad range of unspecific, non-malaria febrile diseases present [3–7]. Therefore, accurate diagnosis becomes a challenge in this complicated epidemiological landscape due to the occurrence of co-infections (e.g. salmonellosis and malaria [8]), lack of accurate, specific and sensitive tools, and inadequately trained personnel.

Within this context, febrile illness infections require different patient management given that a variety of pathogens can cause the diseases. Pathogens include parasites (*Plasmodium*), bacteria (*Salmonella*, *Rickettsia* or *Leptospira*) and viruses (dengue, chikungunya or Zika virus). Thus, the misinterpretation of non-specific fever and the practice of solely empirical diagnoses can lead to inadequate treatment, resulting in high risk of mortality, continued disease transmission and increased antimicrobial resistance [9].

Several state-of-the-art methods are currently being implemented for the diagnosis of fever-related diseases, including microscope examination of stained thick and thin blood smears, and Lateral Flow Tests (LFTs) for malaria [10–12] and non-malaria diseases [13–15].

However, the former may be error-prone due to the subjective interpretation of the results as it depends on the experience of the personnel to see the parasite by eye when using the microscope. The latter, despite being cheap and easy-to-use, also depends on the subjective opinion of the user during the visual read out, especially when the test line of the LFT is not sharply distinguishable. Furthermore, studies have shown that both methods can lack sufficient sensitivity [15–18].

Bacterial and viral culture are also used, but both are labour-intensive, time-consuming and not easy to perform, requiring sophisticated laboratory equipment and qualified staff.

To facilitate accurate species identification, nucleic acid amplification technologies (NAATs), such as PCR or isothermal amplification methods, need to be used [19]. These methods are in general more specific, suitable for a large number of diseases, and have been integrated in some point-of-care (POC) or near-patient systems already on the market or in near-commercialization phase, such as the GeneXpert, FilmArray, ID NOW, cobas Liat, Liaison MDX, GenePOC. Most of them provide various degrees of multiplexity, time-to-result, and integration, as reviewed by Mitsakakis/D’Acremont et al [20], but do not usually cover the malaria/non-malaria febrile illness panel.

In contrast, the VerePLEX Biosystem (Veredus Laboratories) [21] incorporates the VereTrop [22] capable for multi-pathogen detection of tropical diseases. However, it is limited by the high cost of cartridges (100 USD) [23] and the use of two units for diagnosis, one for PCR and hybridization, and one for detection. It requires an *ex situ* sample preparation and the overall time-to-result is 3.5 h. The Q-POC (QuantuMDx) [24] is a portable apparatus at pre-market stage and its first applications are in malaria species subtyping [25] but there is no published evidence for multi-disease detection as yet.

This study addresses the challenges for the technologies and their applications discussed above. We have developed a system for detecting a 12-plex panel of fever-causing pathogens called “FeverDisk”, which is based on the centrifugal microfluidic platform LabDisk [26]. The FeverDisk features fully-automated integrated sample preparation with downstream Loop-mediated isothermal amplification (LAMP) [27, 28], allowing a time-to-result of 70–120 min for the individual assays in a 120 min total run-time window with minimum hands-on time of ~5 min, for pipetting the sample into the cartridge. The selected pathogen panel, whose specific primers were used in the FeverDisk for feasibility demonstration, included: *Plasmodium falciparum (Pf)*, *P*. *vivax (Pv)*, *P*. *ovale (Po)*, *P*. *malariae (Pm)*; *Salmonella enterica* Typhi (ST), *S*. *enterica* Paratyphi A (SPa), *Streptococcus pneumoniae* (Spn), chikungunya virus (CHIKV), dengue virus—serotypes 1–4 (DENV1-4), and Zika virus (ZIKV). The developed assays to detect these pathogens also included the pan-malaria, *Plasmodium* species (*P*spp) assay. This is a non-exhaustive list of fever-related pathogens that could potentially be detected on the platform. The combination of the target pathogens was selected among many others [20] for feasibility demonstration with the criteria to include: (i) the three main types of pathogens, namely, bacteria, viruses and parasites; (ii) vector-borne diseases; and (iii) bacterial diseases that are common in tropical areas and associated with antimicrobial resistance.

Due to the limited access time at the two African sites (both reference centers, suffered from a high workload due to a global Ebola epidemic at the time of the experiments), and consequently the limited number of FeverDisks that could be tested, resulting data was not sufficient for a statistically significant clinical study. Therefore, the goal of this work was (i) the demonstration of the capability of the newly-developed platform and its components to detect as many of the aforementioned pathogens as possible, available in the samples that we had access to; (ii) the proof-of-principle of fully automated sample-to-answer operation with minimum hands-on time; and (iii) the analytical performance characterization of the platform. A first test series was conducted at the Institut Pasteur in Dakar (IPD), Senegal (West Africa), followed by a second test series at the Central Laboratory in Khartoum (CLK), Sudan (East Africa). In both cases, biobanked samples were analyzed using the FeverDisk and were compared to reference methods available at each site. Some bacterial pathogens which were not available in biobanked samples, were used as cultured samples for specificity assessment (in Dakar), and with varying spiked concentrations for limit of detection assessment (in the third small-scale study in Reinfeld, Germany).

## Materials and methods

### Ethics statement

For the test series in Senegal and Sudan, samples used in the studies were part of the collections of Institut Pasteur in Dakar and Central Laboratory Khartoum, respectively. Ethical approval for retrospective use of the anonymized samples (acquired under informed consent) in diagnostic development research was available from the Ministry of Health of Senegal, as well as from the Central Laboratory Ethical Committee and the Ministry of Higher Education and Scientific Research in Sudan.

### Sample cohorts and reference methods

Samples for the Senegal test series were acquired from retrospective studies and experiments were carried out at the Virology Department of IPD, which is a WHO Collaborating Center for Arboviruses and Haemorrhagic Fever Reference and Research. In particular:

The CHIKV samples (serum) were collected during the Senegal epidemic of 2015 in Kedougou (southeastern region). They were stored at -80°C and were tested at IPD by benchtop real-time RT-PCR [29] and real-time RT-LAMP [30] as reference methods.

The DENV samples (supernatant of infected cells) were collected during epidemics outbreaks in Senegal (2014) and Mauritania (2014). They were stored at -80°C and were tested at IPD by benchtop real-time RT-PCR [31] and real-time RT-LAMP [32] as reference methods.

The malaria samples (blood) were collected in Kedougou, as part of the local entomological and human serosurveys program of febrile illness diseases, including malaria and main arbovirus diseases. The program was dedicated to systematic surveillance of malaria, dengue and chikungunya fever. These samples were aliquoted and stored at +4°C for malaria tests. Reference tests were done by microscopy, LFTs and PCR. For the latter, more details are available in Niang et al [33].

The *Salmonella* Typhi and Paratyphi A samples were stool bacterial cultures and were tested for the purpose of specificity assessment against the other pathogens’ primers of the FeverDisk. Reference tests were done by standard microbiology diagnostic procedures, namely culture, isolation, identification and antimicrobial susceptibility testing, according to the ISO 6579 standard. The extracted DNA using the QIAamp DNA Mini Kit, QIAGEN, Germany was quantified using the Nanodrop spectrophotometer instrument at the Bacteriology Department of IPD. Further information on the preparation of the cultured samples are provided in section A in S1 Text.

Malaria samples (whole blood) for the Sudan test series were acquired from retrospective studies and experiments were carried out at CLK. Samples were stored at -20°C before testing. All samples were additionally tested with the reference methods available on site, i.e. microscopic examination of thin and thick blood smears as well as benchtop LAMP.

In addition, during the test period, one patient presented with acute fever, sample (whole blood) was collected in real-time following the required ethics conditions, and the sample was tested immediately on site.

### Nucleic acid extraction

Total nucleic acid extraction from raw samples was performed using a solid-phase extraction kit based on Boom chemistry [34].

For the reference benchtop RT-PCR and RT-LAMP of the biobanked DENV and CHIKV samples in IPD, RNA extractions were carried out using the QIAamp Viral RNA Mini Kit (QIAGEN, Germany) following the supplier’s recommended protocol.

For the reference benchtop PCR of the biobanked malaria samples in IPD, genomic DNA extraction of *Plasmodium* parasites was performed using the QIAmp DNA Mini Kit (QIAGEN, Germany) according to supplier’s protocol.

For the reference benchtop LAMP of the biobanked samples in CLK, DNA was extracted using innuPREP MP Basic Kit A (art. # 845-KS-4900500, Analytik Jena GmbH, Germany).

### Total nucleic acid extraction on the FeverDisk

For the FeverDisk experiments, silica coated magnetic beads (MagSuspension, from innuPREP MP Basic Kit A, # 845-KS-4900500, Analytik Jena GmbH, Germany) were pre-stored in dry format on the FeverDisk. The beads were combined with nucleic acid extraction buffers (MagnaMedics Diagnostics BV, currently magtivio BV, the Netherlands), pre-stored in the FeverDisk in stick-packs [35]. The custom-developed extraction protocol, which was automated on the FeverDisk, was also performed manually as described in section B in S1 Text.

### Protocol for manual nucleic acid amplification

For the real-time PCR based benchtop reference amplification of the malaria biobanked samples in IPD, *Plasmodium* spp detection as well as differentiation from CHIKV, DENV, YFV and ZIKV was performed using multiplex real-time amplification and a nested PCR protocol as previously described by Niang et al [33].

For the real-time RT-PCR based benchtop reference amplification of the CHIKV and DENV biobanked samples at IPD, the protocols are described in Pastorino et al [29] and Wu et al [31], respectively.

For the real-time RT-LAMP based benchtop reference amplification of the CHIKV and DENV biobanked samples at IPD, RT-LAMP reagents (except primers) were the same for all reactions. Total reaction volume was 25 μL for all reactions. RT-LAMP reactions were run at 64°C using an ABI7500 Fast real-time PCR system (Applied Biosystems) according to Lopez-Jimena et al [30,32]. All the tube assays conducted at the IPD were performed in triplicates, with the exception of the sample 274755 that was tested in duplicates for DENV4.

For the real-time LAMP based benchtop reference amplification of the malaria biobanked samples in CLK, LAMP was performed in 25 μL for all reactions including all reagents, primer sets and template. Reactions were run at 64°C using a Rotor-gene cycler (QIAGEN, Germany) and amplification time was 1 h.

For the real-time nucleic acid amplification on all the FeverDisk experiments, all reagents needed for the LAMP amplification stage were pre-stored on the FeverDisk in lyophilized form. Primers used for each target were dried in each reaction chamber. Lyophilized pellets were developed and provided by MAST Group Ltd, UK.

### LAMP primer design

Primer sets for the CHIKV and DENV1-4 virus targets were designed using a combination of Principal Component Analysis (PCA) and LAMP primer design based on sequence alignments using the LAVA software [36]. DENV primer sets were based on full genome sequences whilst CHIKV primer sets were designed on the same target region (6K-E1 region) [30,32].

Two different ZIKV assays were tested based on different primer design approaches [37]. One of the assays was developed following the methodology described by Lopez-Jimena et al [32]. Briefly, it consisted of an open source approach, in which 45 whole genome ZIKV sequences, covering strains from 1947 to 2015–2016, were retrieved from NCBI database, submitted to phylogenetic analysis, principal component analysis (PCA) and primer design using LAVA software. The second developed ZIKV assay was based on standard software for LAMP primer design (Primer Explorer V4), using 59 sequences of the ZIKV 3’ untranslated region (UTR). Further *in silico* tests using VisualOMP (DNA software) discarded primer sets based on primer dimerization and secondary structures.

For *Plasmodium*, *Salmonella enterica* Typhi and Paratyphi A, as well as *Streptococcus pneumoniae* (all designed at Mast Diagnostica GmbH), primer sets targeting specific regions of the genome (18s and/or mitochondria for *Plasmodium* species, STY0201 for *S*. Typhi, SSPA2308 for *S*. Paratyphi A, and lytA and PsaA for *S*. *pneumoniae*) were designed using Primer Explorer (http://primerexplorer.jp/e/).

### Sample-to-answer operation of the FeverDisk and corresponding processing device

**Fig 1:**
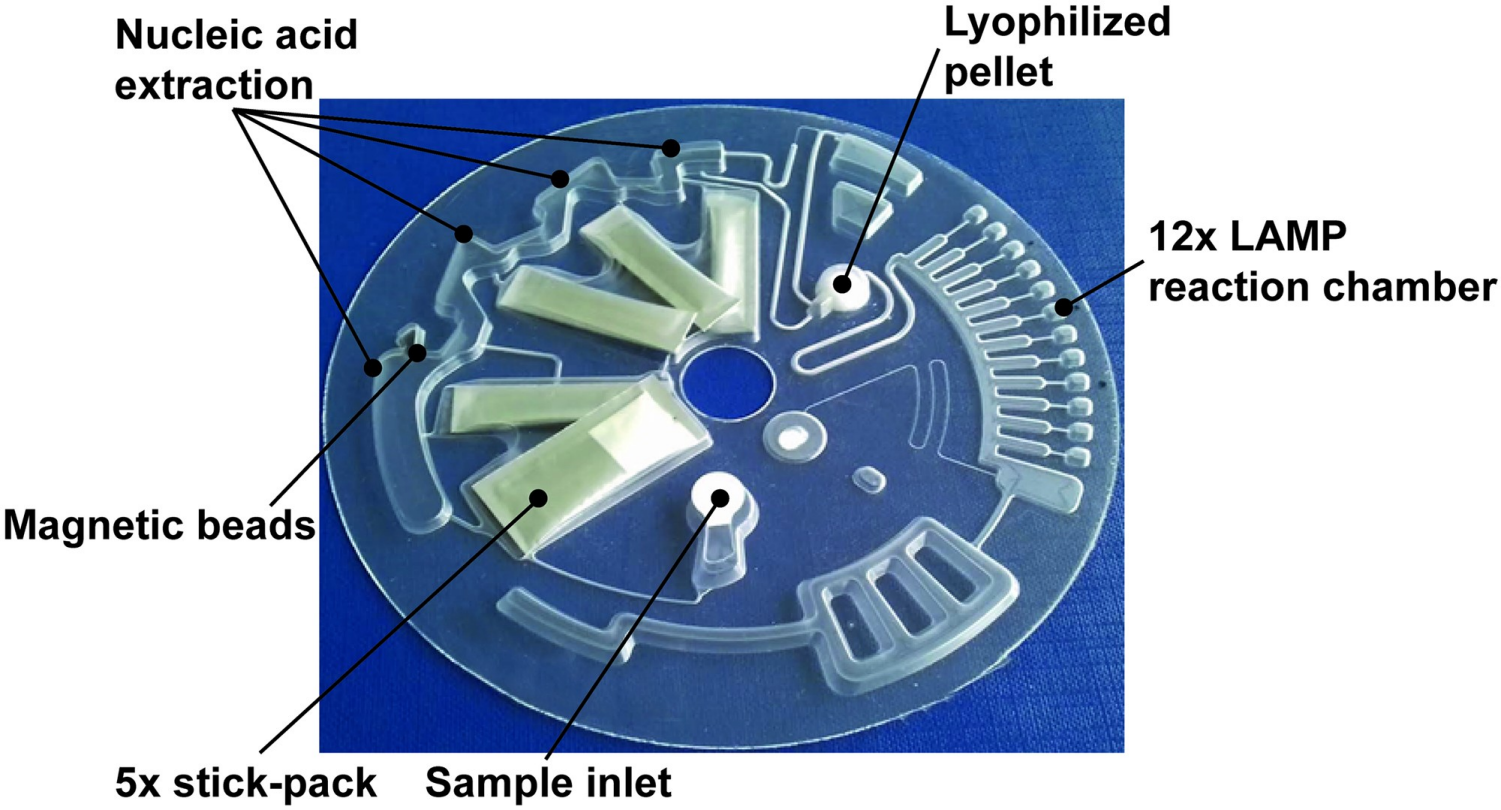
Photograph of a FeverDisk cartridge. All reagents are pre-stored for fully automated nucleic acid analysis. The sample inlet is sealed with a pre-mounted sealing foil.

**Fig 2:**
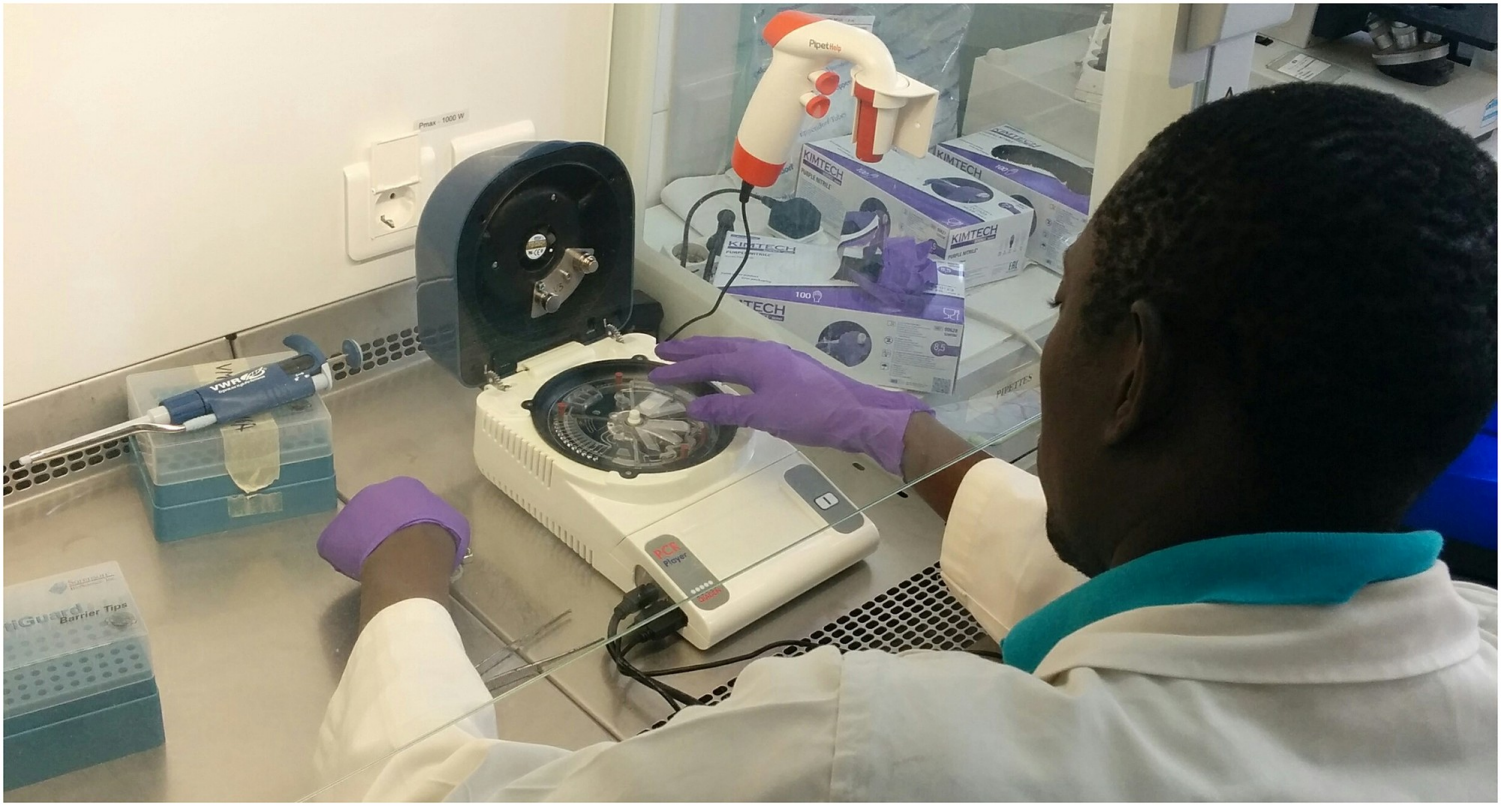
Photograph of the FeverDisk processing device. Dr. Cheikh Fall, co-author from the Institut Pasteur de Dakar, prepares a FeverDisk for measurement.

For FeverDisk operation, the cartridge is placed into a LabDisk player 1 (ESMO01-MB-3044, QIAGEN Lake Constance, Germany). The device features a customized holder for cartridge fixation, stationary magnets for magnetic bead manipulation, as well as temperature and rotational frequency control for microfluidic processing as presented in prior work [38]. In the present study, fluorescence detectors allowed for detection of nucleic acid amplification product in the green channel (FAM). The FeverDisk (Fig 1) can handle a sample volume of 200 μL. The only manual steps to complete are sample addition and sealing of the inlet with a pressure-sensitive adhesive foil. The LabDisk player 1 (Fig 2) executes the required operations in a fully automated way until result acquisition. In short, this includes: Sample lysis, nucleic acid binding to magnetic beads, two times washing of the nucleic acids followed by an elution step. After nucleic acid extraction, the obtained eluate is mixed with lyophilized amplification reagents (enzymes, salts, dNTPs, dye). Then, this mastermix is aliquoted into twelve reaction chambers of 10 μL volume, each. Last, nucleic acids are amplified by LAMP and signal is acquired in the green channel. Centrifugal microfluidics is advantageous in the present application, since it provides liquid control by centrifugal force. The resulting fully automated analysis can be achieved without external pumps or tubes, for liquid handling. The system builds up on so-called microfluidic unit operations [26,39]. Some advanced centrifugal microfluidic unit operations that are used in this work in order to enable full automation are: (i) magnetic bead transfer under continuous rotation during the extraction [40]; (ii) monolithic valves using temperature change-rate (TCR) actuated valving [41]; (iii) centrifugo-dynamic inward pumping [42]; and (iv) dead-volume free and monolithic rehydration and mixing of the lyophilized LAMP reagent pellet using TCR-actuated bubble-mixing [43]. More details on the microfluidic and temperature protocols and sequence of steps are given in S1 Fig and S1 Table. Details on the FeverDisk production are given in section C in S1 Text. Experiments were performed on three devices at three different locations, namely in Dakar (Senegal), Khartoum (Sudan), and Reinfeld (Germany).

### Data analysis

Fluorescence signals from FeverDisk experiments were analyzed to determine the time-to-positive (*T*_*p*_) of the LAMP reactions using a custom script (Mathematica, Wolfram Research, Inc., UK) and following the procedures for data normalization and *T*_*p*_ calculation described in our prior work [43]. *T*_*p*_ was defined as the time, where the normalized fluorescence signal reached a threshold level of 20 standard deviations above the baseline allowing to reliably filter possible noisy background. As control for microfluidic integrity, i.e. filling of read-out chambers, the baseline signals of the raw data from FeverDisk experiments were required to be larger than 200 mV.

## Results

### FeverDisk results of the test series in Dakar, Senegal

The known positive biobanked samples were tested using either one or two FeverDisks per sample, depending on the volume availability. An overview of disks and results is shown in Table 1. The detailed results are provided in the following sections.

**Table 1:** Overview of results from the test series in Senegal where 29 FeverDisks were used. The reference methods as well as the FeverDisk results are shown.

Specimen details				Reference methods					FeverDisk test results in Senegal		
ID ^a^	Origin	Confirmed/Expected pathogen/ infection	Sample	#1 Benchtop (RT)-PCR (Ct)	#2 Benchtop (RT)-LAMP (*T*_*p*_ in min)^b^	#3 Microscopy (pos/neg)	#4 LFT (pos/neg)	#5 Culture & DNA quantification (ng/μL)	Pathogens detected	*T*_*p*_ (in min)	Result
274755-A	Senegal, 2015	CHIKV DENV4	Serum	CHKV: 25.30 DENV4: n/a ^c^	CHIKV: 16–17 DENV4: 35–40 (2/2)	n/a n/a	n/a n/a	n/a n/a	CHIKV DENV4	32 45	TP TP
274754-B	Senegal, 2015	CHIKV DENV2	150 μL serum + 50 μL H_2_O ^d^	CHIKV: 25.13 DENV2: n/a	CHIKV: 16–17 DENV2: 45–50 (1/3)	n/a n/a	n/a n/a	n/a n/a	CHIKV DENV2	27 >65	TP TP
274443-A	Senegal, 2015	Malaria CHIKV	Serum	*P*spp: 35.82 CHIKV: neg	*P*spp, *Pf*: n/a CHIKV: 30–35 (2/3)	n/a	n/a	n/a	*P*spp *Pf* CHIKV	29 40 -	TP TP TN
274443-B									*P*spp *Pf* CHIKV	23 33 -	TP TP TN
274438-A	Senegal, 2015	CHIKV	Serum	29.28	CHIKV: 18–19	n/a	n/a	n/a	-	-	FN
274438-B									-	-	FN
274530-A	Senegal, 2015	CHIKV	150 μL serum + 50 μL H_2_O ^d^	33.22	CHIKV: 16–17	n/a	n/a	n/a	-	-	FN
267197-A	Senegal, 2014	DENV ^e^	Supernatant of infected cells	25.89	DENV1: 20	n/a	n/a	n/a	DENV1	41	TP
267175-B	Maurita nia, 2014	DENV ^e^	Supernatant of infected cells	29.79	DENV1: 21–22	n/a	n/a	n/a	DENV1	45	TP
267267-A	Senegal, 2014	DENV2	Supernatant of infected cells	27.82	DENV2: 30–31	n/a	n/a	n/a	DENV2	64	TP
267267-B									DENV2	62	TP
267150-A	Senegal, 2014	DENV2	Supernatant of infected cells	26.15	DENV2: 28–29	n/a	n/a	n/a	DENV2	-	FN
267150-B									DENV2	59	TP
267150-C									DENV2	-	FN
267207-A	Senegal, 2014	DENV2	Supernatant of infected cells	38.48	DENV2: 39–45	n/a	n/a	n/a	DENV2	-	FN
C71-A	Senegal, 2016	Malaria (*P*. *falciparum*)	150 μL blood + 50 μL PBS ^f^	*Pf*: 31.16	*Pf*: n/a	pos	pos	n/a	*P*spp *Pf*	22 26	TP TP
C71-B									*P*spp *Pf*	18 25	TP TP
C74-A	Senegal, 2016	Malaria (*P*. *falciparum*)	Blood	*Pf*: 24.91	*Pf*: n/a	pos	pos	n/a	*P*spp *Pf*	18 25	TP TP
C74-B									*P*spp *Pf*	29 40	TP TP
B32-B	Senegal, 2016	Malaria (*P*. *falciparum*)	150 μL blood + 50 μL PBS ^f^	*Pf*: 26.47	*Pf*: n/a	n/a	neg	n/a	*P*spp *Pf*	- -	FN FN
S.12-A	IPD Clinical Lab	*S*. Typhi	Stool culture	n/a	*S*. Typhi: n/a	n/a	n/a	109.9	*S*. Typhi	22	TP
S.12-B									*S*. Typhi	23	TP
S.15-A	IPD Clinical Lab	*S*. Typhi	Stool culture	n/a	*S*. Typhi: n/a	n/a	n/a	59.4	*S*. Typhi	22	TP
S.16-A	IPD Clinical Lab	*S*. Typhi	Stool culture	n/a	*S*. Typhi: n/a	n/a	n/a	205.9	*S*. Typhi	15	TP
S.16-B									*S*. Typhi	22	TP
S.pT1-A	IPD Clinical Lab	*S*. Paratyphi A	Stool culture	n/a	*S*. Paratyphi A: n/a	n/a	n/a	116.2	*S*. Paratyphi A	31	TP
S.pT1-B									*S*. Paratyphi A	37	TP
S.pT3-A	IPD Clinical Lab	*S*. Paratyphi A	Stool culture	n/a	*S*. Paratyphi A: n/a	n/a	n/a	395.0	*S*. Paratyphi A	53	TP
S.pT4-A	IPD Clinical Lab	*S*. Paratyphi A	Stool culture	n/a	*S*. Paratyphi A: n/a	n/a	n/a	287.4	*S*. Paratyphi A	44	TP

^a^: The letters “A”, “B”, “C” next to the sample ID mean that there was one or more disks, respectively, tested with sample from the same vial.

^b^: Time-to-positive (*T*_*p*_) ranges of benchtop LAMP results, representing triplicates, except 274755 that was tested in duplicate for DENV4. Between brackets there is information about positive results obtained within the total of replicates.

^c^: Method not available.

^d^: Due to lack of sufficient material to run two disks, 50 μL molecular grade H_2_O were added to the 150 μL of sample.

^e^: Serotype determined by the FeverDisk and confirmed by the RT-LAMP.

^f^: The sample was deemed clotted and thus diluted with a small amount of PBS.

#### Detection of co-infections

**Fig 3:**
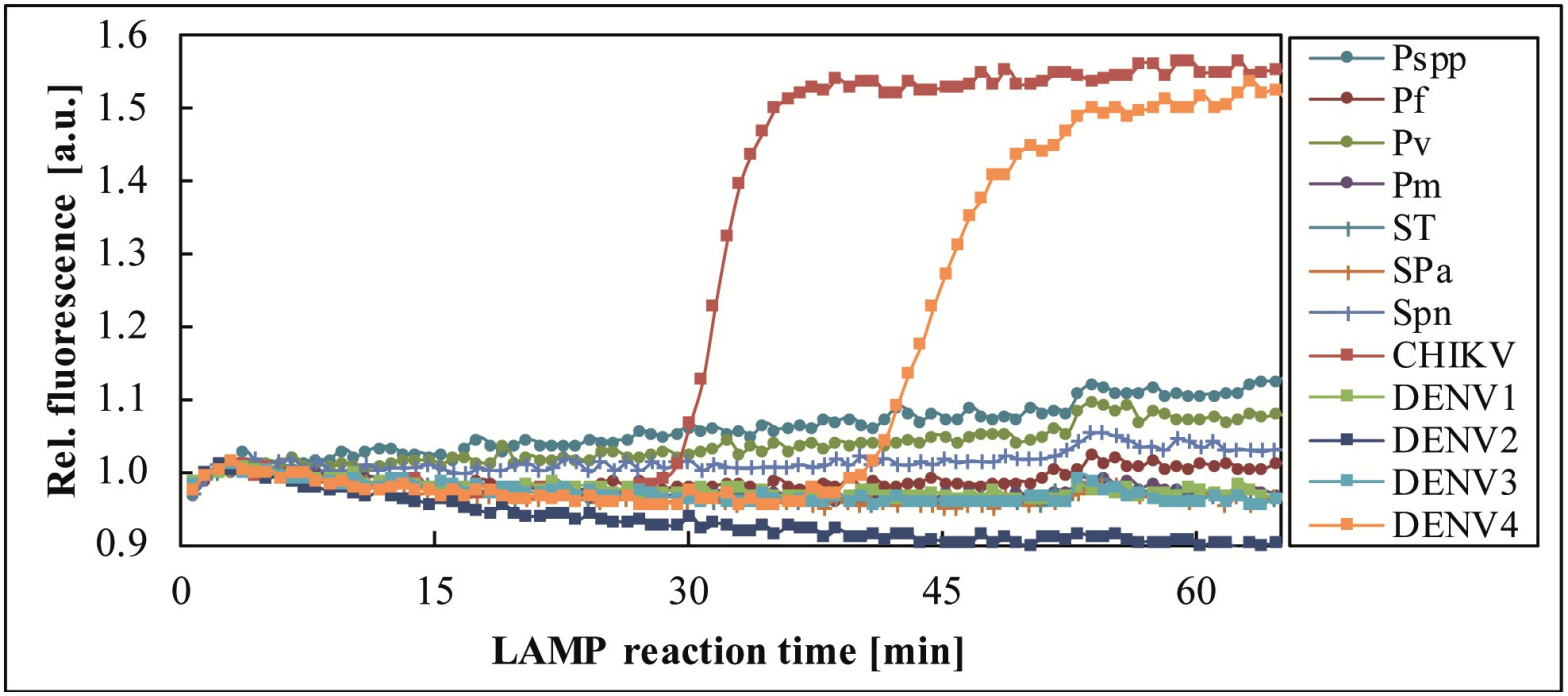
Detection of CHIKV and DENV4 co-infection in sample 274755. The FeverDisk showed a true positive DENV4 amplification in addition to the CHIKV-expected amplification.

Sample 274755, collected during a CHIKV epidemic in 2015, CHIKV positive by benchtop real-time RT-PCR (Ct 25.30) and real-time RT-LAMP (*T*_*p*_ 16–17 min), was tested by one FeverDisk and yielded a true positive CHIKV signal, as well as an additional positive signal for DENV4 in chamber 12 (Fig 3). A subsequent benchtop real-time RT-LAMP confirmed DENV4 (2/2) in this sample, which was therefore considered a true positive co-infection of CHIKV and DENV4.

**Fig 4:**
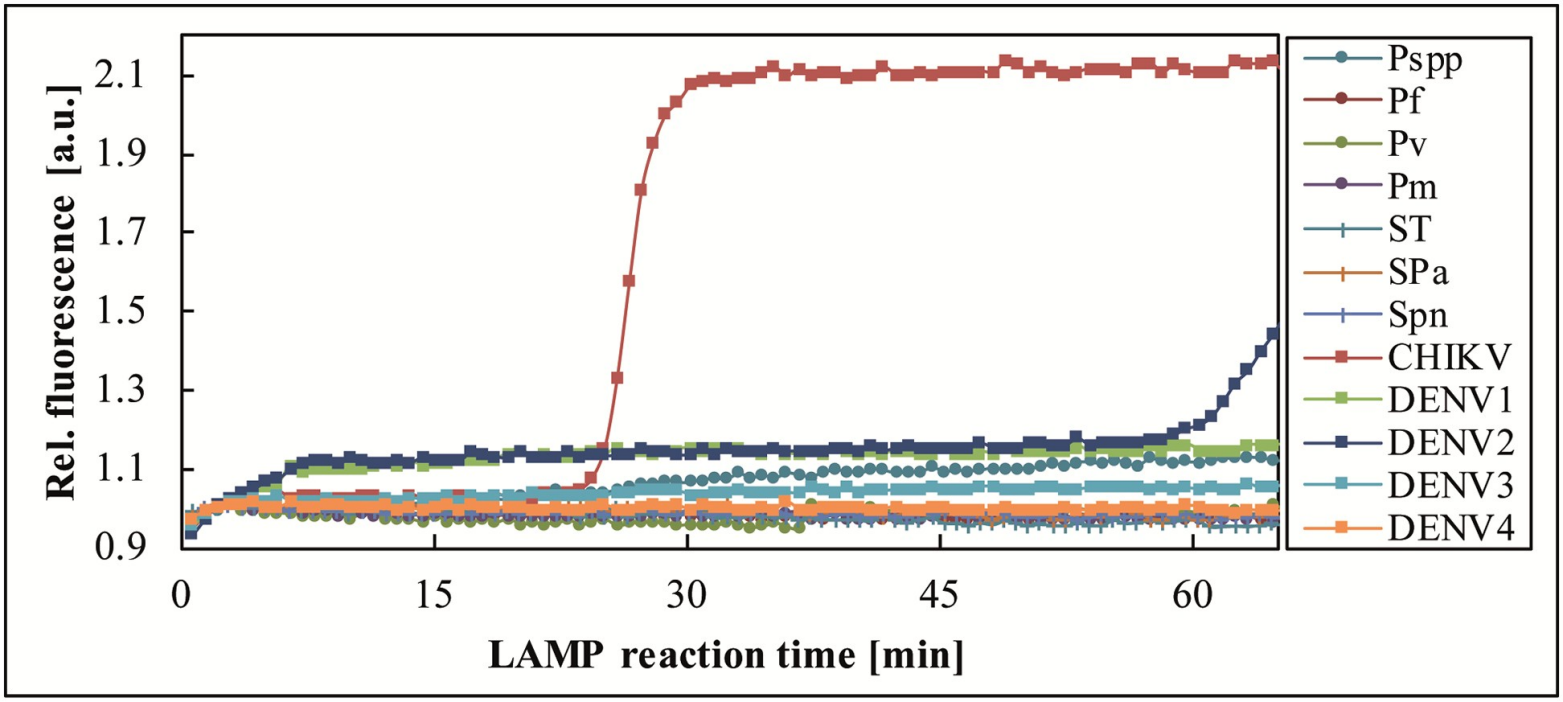
Detection of CHIKV and DENV2 co-infection in sample 274754. The FeverDisk showed a true positive DENV2 amplification in addition to the CHIKV-expected amplification.

Sample 274754, CHIKV positive by benchtop real-time RT-PCR (Ct 25.13) and real-time RT-LAMP (*T*_*p*_ 16–17 min) was tested by one FeverDisk, which confirmed CHIKV presence and yielded an additional amplification signal for DENV2 in chamber 10 (Fig 4). To verify the DENV2 signal, the sample was tested in triplicate for DENV2 by benchtop real-time RT-LAMP, which yielded 1/3 positives. Considering that the sample was diluted before use on the FeverDisk (Table 1, 150 μL serum + 50 μL molecular grade H_2_O, instead of 200 μL serum, due to lack of sufficient volume), the results from both, the benchtop and the FeverDisk LAMP indicate that DENV2 co-infection was detected close to the limit of detection (LOD).

Sample 274443 was CHIKV positive by real-time RT-PCR (Ct 32.80) at the time of collection. When tested with two FeverDisks it provided CHIKV negative results (S2 Fig). Subsequent repetition of the real-time RT-PCR six months after collection (at the time of FeverDisk experiments) yielded negative results, confirming the FeverDisk results. Most likely the viral RNA in the sample had degraded. The benchtop real-time RT-LAMP gave positive amplification between 30–35 min (2/3 repetitions), which indicates that the RT-LAMP managed to amplify remaining non-degraded viral RNA, while being at the limit of detection, and possibly being slightly more sensitive than the LAMP in FeverDisk, which explains why there was no amplification by RT-PCR or the FeverDisk. In addition, this sample was found positive for *Pf* and *P*spp in both FeverDisk runs (S2 Fig). A subsequent benchtop malaria real-time PCR for *P*spp yielded a Ct value of 35.82, which confirmed the FeverDisk results.

#### Determination of unknown DENV serotype in biobanked samples

**Fig 5:**
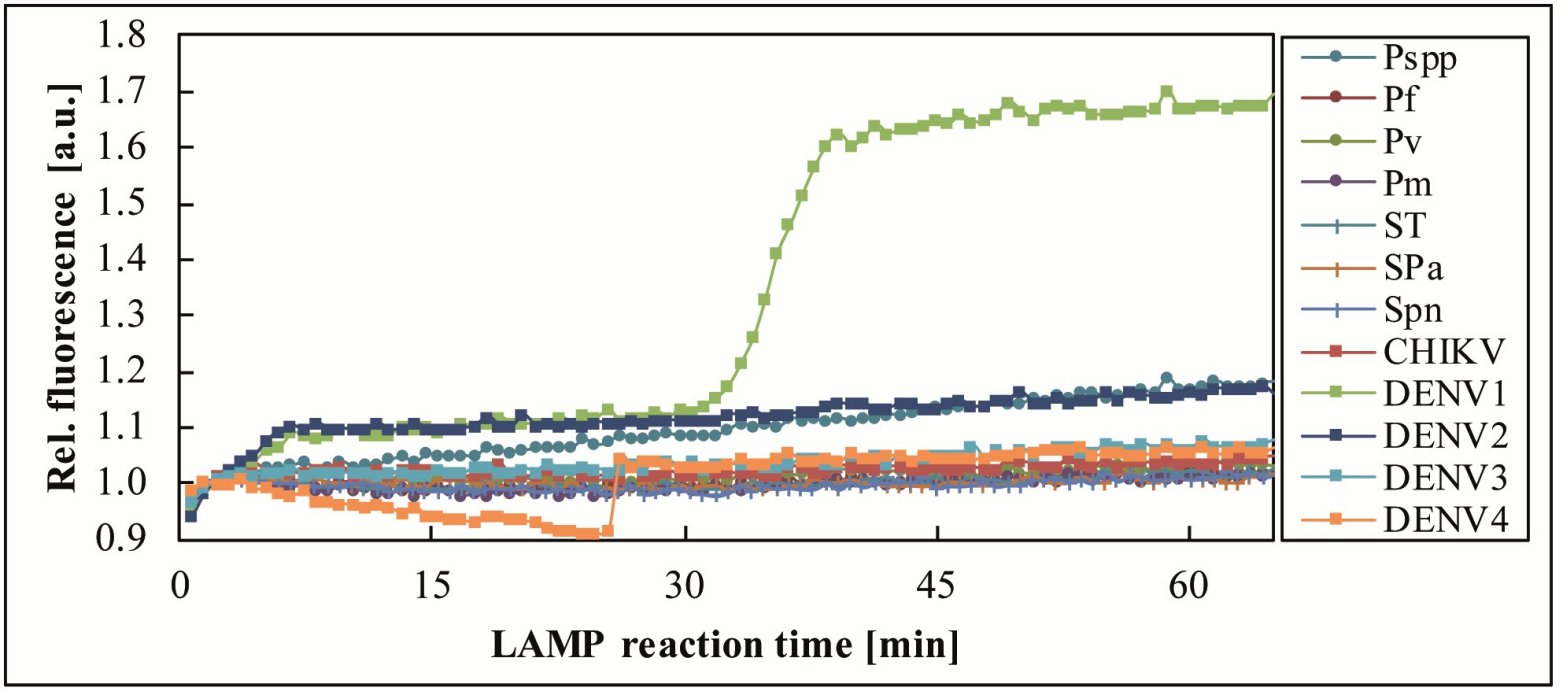
Detection of DENV and determination of initially unknown serotype. Sample 267197 is shown here; sample 267175 showed similar results.

Two samples, 267197 and 267175 were characterized with benchtop RT-PCR as DENV positive (Ct 25.89 and 29.79, respectively) but of unknown serotype and were tested with one FeverDisk each. The FeverDisk panel allowed to determine that both DENV samples were of serotype 1, as the amplification signal appeared in chamber 9 where DENV1 primers were pre-stored (Fig 5). Serotyping was confirmed by benchtop RT-LAMP [32].

#### Detection of single infectious pathogens

Several samples yielded FeverDisk results for a single infectious agent (S3 Fig) and confirmed their corresponding reference method results (Table 1).

Sample 267267, DENV2 positive by benchtop real-time RT-PCR (Ct 27.82) and real-time RT-LAMP (*T*_*p*_ 30–31 min), was tested with two FeverDisks, both confirming this result as true positive.

Sample 267150, DENV2 positive by benchtop real-time RT-PCR (Ct 26.15) and real-time RT-LAMP (*T*_*p*_ 28–29 min) was positive in 1/3 FeverDisks and was considered positive close to the limit of detection of the method.

Samples C71 and C74 were malaria *(P*. *falciparum)* positive by benchtop PCR (Ct 31.16 and 24.91, respectively). Two FeverDisks were used for each sample, and all four were true positive for *P*spp and *Pf*.

Samples S.12, S.15, S.16 and samples S.pT1, S.pT3, S.pT4 were provided as *Salmonella* Typhi and *Salmonella* Paratyphi A stool cultures, respectively. Reference results were produced by culture followed by DNA quantification using the Nanodrop spectrophotometer. All FeverDisks that were used with these samples confirmed the presence of the pathogen. Although these were not clinical samples, we provide the results as additional data sets for the successful fully automated FeverDisk operation and for the specificity of the other primers of the FeverDisk against the *Salmonella* samples.

#### False negative and negative results

Samples 274438 and 274530, CHIKV positive by benchtop real-time RT-PCR (Ct 29.28, Ct 33.22) and real-time RT-LAMP (*T*_*p*_ 18–19 min, *T*_*p*_ 16–17 min), were not confirmed by two and one FeverDisks, respectively, which were considered to be false-negative results. However, due to limited available volume in sample 274530, instead of 200 μL sample we added 50 μL molecular grade H_2_O to the available 150 μL sample, which may have contributed to the negative result.

Sample B32 was tested with two reference methods and was found *P*. *falciparum* positive by PCR (Ct 26.47) but negative by LFT (microscopy test had not been performed). LFTs have typically lower sensitivity than the PCR, therefore LFT was considered false negative. A negative result in the one FeverDisk used was considered false negative, however again a dilution of the sample had to be done as described above.

Sample 267207, DENV2 positive by benchtop real-time RT-PCR (Ct 38.48) and real-time RT-LAMP (*T*_*p*_ 39–45 min) was negative in the one FeverDisk that was used. Considering the high Ct and *T*_*p*_ values of the reference methods, it can be assumed that this sample was false negative by FeverDisk because the RNA concentration was beyond the limit of detection of the method.

### FeverDisk results of the test series in Khartoum, Sudan

A total of eight samples were made available during the Sudan test series (Table 2).

**Table 2:** Overview of results from the test series in Sudan where 9 FeverDisks were used. The reference methods as well as the FeverDisk results are shown.

Specimen details				Reference methods		FeverDisk test results in Sudan		
ID ^a^	Origin	Confirmed/Expected pathogen/infection	Sample	#1: Benchtop LAMP (*T*_*p*_ in min) ^b^	#2: Microscopy (pos/neg)	Pathogens detected	*T*_*p*_ (in min)	Result
#5	CLK	Malaria	Blood	*P*spp: 6 *Pf*: 10	pos pos	*P*spp *Pf*	13 15	TP TP
#6	CLK	Malaria	Blood	*P*spp: 5 *Pf*: 7	pos pos	*P*spp *Pf*	19 25	TP TP
#13	CLK	Malaria	Blood	*P*spp: 5 *Pf*: 7	pos pos	*P*spp *Pf*	13 17	TP TP
#14-A	CLK	Malaria	Blood	*P*spp: 7 *Pf*: 9	pos pos	*P*spp *Pf*	8 9	TP TP
#14-B						*P*spp *Pf*	13 15	TP TP
#20	CLK	Malaria	Blood	*P*spp: 7 *Pf*: 54	pos pos	*P*spp *Pf*	13 16	TP TP
#36	CLK	Malaria	Blood	*P*spp: 10 *Pf*: 12	neg neg	*P*spp *Pf*	11 13	TP TP
#62	CLK	Malaria	Blood	neg	neg	*P*spp *Pf* *Pv*	- - -	TN TN TN
#X^c^	CLK	Malaria	Blood	n/a	pos	*P*spp *Pf*	11 13	TP TP

^a^: “A”, “B” next to the patient ID means that there was one or two disks, respectively, tested with sample from the same vial.

^b^; Time-to-positive (*T*_*p*_) of LAMP results.

^c^: This is the fresh sample collected in real-time upon presence of a patient suspected with malaria after clinical examination.

Samples #5, #6, #13, #14 and #20, positive by benchtop LAMP and microscopy, were tested by one FeverDisk (sample #14 by two) and yielded true positive *P*spp and *Pf* signals.

Sample #36 was malaria positive by benchtop LAMP but negative in microscopy. Contradictory results between the two reference methods are due to the different sensitivities. Nucleic acid amplification techniques have a higher sensitivity than microscopy. Also, microscopy is based on visual inspection and depends on experience and subjectivity of the personnel. Therefore, the one FeverDisk that was tested and yielded positive results for *P*spp and *Pf*, is considered true positive because it is in agreement with the more sensitive benchtop LAMP.

Sample #62, negative by benchtop LAMP and microscopy, yielded a negative signal in all tested *Plasmodium* assays (*P*spp, *Pf*, *Pv*) present in the panel of the one FeverDisk that was tested. This is considered true negative as it is in accordance with the more sensitive among the two reference methods (see comment above).

**Fig 6:**
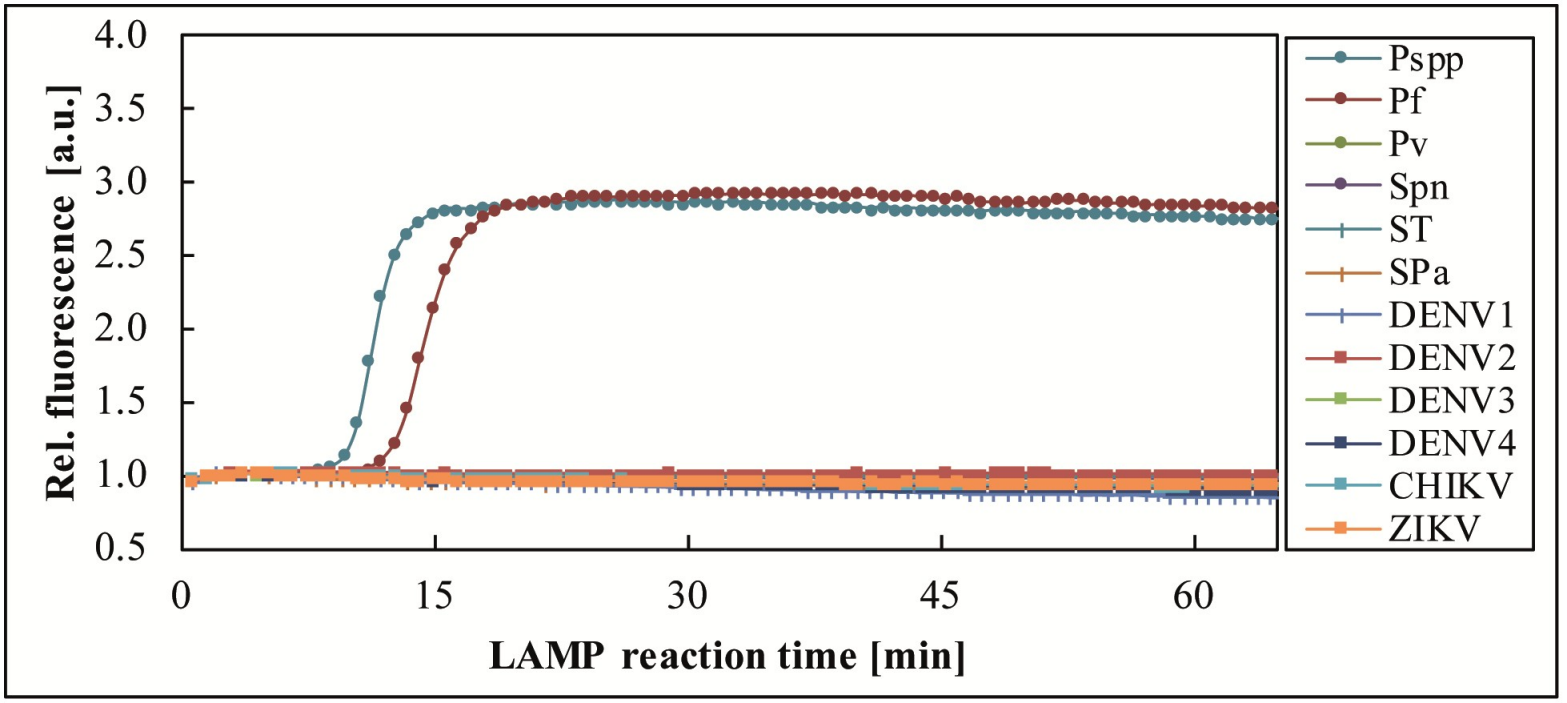
Detection of malaria on a fresh whole blood sample collected in real time. Positive signal for *Pf* and *P*spp was confirmed as true positive with microscopy blood smear test.

During the days of the tests, one patient presented with acute fever, and after clinical examination was found *Pf* positive based on microscopy. A blood sample (coded as “Sample X”, Table 2) was taken and immediately tested in the FeverDisk, which yielded *Pf* and *P*spp positive results (Fig 6).

### FeverDisk results of the test series in Reinfeld, Germany

Given the limited availability of samples and no presence of bacteria in the biobanked samples (except for the cultured samples), the authors performed a laboratory assessment of the analytical sensitivity of *S*. *pneumoniae*, *S*. Typhi and *S*. Paratyphi A by simultaneously spiking known concentrations in 200 μL whole blood (see Table 3 and S4 Fig for the real-time amplification curves). Twelve FeverDisks were used for *S*. Typhi, *S*. Paratyphi A and *S*. *pneumoniae*, with 3 additional FeverDisks used for *S*. Paratyphi A only. The LOD with a 95% probability for the three examined bacteria was calculated based on probit analysis, using Minitab 17 statistical software (Minitab Inc). The LOD for *S*. *pneumoniae*, *S*. Typhi and *S*. Paratyphi A was calculated 1394 CFU/mL, 1959 CFU/mL, and 25546 CFU/mL, respectively. The one order of magnitude difference in the LOD of *S*. Paratyphi A is attributed to comparatively lower sensitivity of the assay (see Table 3). The analytical sensitivity of the FeverDisk for *P*. *falciparum* and the viral assays was not determined due to the insufficient resources and number of FeverDisks.

**Table 3:** Bacterial concentrations tested in the FeverDisk to calculate the limit of detection and the ratio of positive results within the total repetitions assayed.

Bacteria	CFU/mL	Positive results/repetitions
*S*. Paratyphi (serotype A)	2×10^4^	8/8
	1×10^4^	11/16
	5×10^3^	9/16
*S*. Typhi	5×10^3^	8/8
	2.5×10^3^	7/8
	1×10^3^	12/12
	5×10^2^	9/12
*S*. *pneumoniae* (serotypes 3 and 5)	5×10^3^	8/8
	2.5×10^3^	8/8
	1×10^3^	10/12
	5×10^2^	4/12

## Discussion

The need to cover the major differentials for fever of unknown origin in a tropical to subtropical setting is a daunting task, which is made even more challenging due to infrastructure issues such as unstable provision of electricity or lack of refrigeration equipment for storage of diagnostic tests. In order to integrate sensitive, specific, rapid sample-to-answer testing of an essential panel of differentials in a user friendly way, the FeverDisk platform uses a microfluidic disk to extract the total nucleic acid from samples in order to screen for 12 individual parameters simultaneously in multiple distinct reaction chambers and using a point-of-care compatible device. As our results show, this can lead to multiple detections and confirm co-infections within a maximum of 2 h after the start of the automated analysis. The advantage is that the individual reactions do not compete with one another and full analytical sensitivity is therefore available for each parameter. The detection concept is qualitative (positive/negative output) and the *T*_*p*_ value does not quantitatively correlate to the viral/bacterial/parasitic load. The goal of the experiments was to demonstrate the proof-of-principle of the platform in operational settings, with biobanked samples, as well as to assess the analytical performance. The rationale behind the selection of the target pathogens was that: (i) they represent some of the main fever-causing agents [20]; (ii) they cover three types of pathogens, namely parasites, bacteria and viruses, therefore their differentiation is of crucial clinical utility and impact for downstream treatment; and (iii) DENV, CHIKV and ZIKV cover arboviruses co-circulating in the same epidemic areas as malaria contributing to fever of unknown origin in Africa, but also in Latin America and South East Asia [44–47].

Given that each FeverDisk integrates 12 assays, we characterize its performance on the assay level. Thirty-eight biobanked samples tested by FeverDisk in both test series provided 59 assay results (40 in Senegal, 19 in Sudan; *P*spp and *Pf* counted as individual assays, see Tables 1 and 2) out of which 51 were confirmed and in agreement with reference methods (32/40 in Senegal and 19/19 in Sudan). This yields an 86.4% agreement between the FeverDisk- and reference method-confirmed assays. These results are sufficient to indicate the potential of the platform.

### Analytical performance of the FeverDisk

Each FeverDisk provides 12 LAMP assay results. Each sample containing a targeted pathogen tested in a FeverDisk is expected to give at least one positive result from a chamber containing the respective specific primers and up to 11 negative results, provided that the targeted organism concentration is within the detection limits of the respective assay. As shown in the test series in Senegal, in some cases detection of co-infections are possible.

In this study, FeverDisk results were characterized as follows in comparison to reference tests:

True positive (TP): positive in disk; confirmed positive by reference method
True negative (TN): negative in disk; confirmed negative by reference method
False positive (FP): positive in disk; negative by reference method
False negative (FN): negative in disk; positive by reference method
Negative (N): negative in disk; not tested by reference method
Case N refers to all (up to 12) negative results per FeverDisk, for which reference methods were not done to verify the negativity, therefore they are characterized as “negative” and not as “true negative”.

All positive FeverDisk results were confirmed by reference test results (Tables 1 and 2). Mostly, more than one reference test was used and a considerable number of samples were confirmed retrospectively after FeverDisk testing.

Interestingly, there were two cases that exhibited discrepancy between the reference methods. The Senegal sample B32, malaria positive by benchtop PCR, was LFT negative and FeverDisk negative. The Sudan sample #36, malaria positive by benchtop LAMP, was negative by microscopy and FeverDisk positive.

In both cases, the molecular reference method, real-time PCR or real-time LAMP, was more sensitive [48], and the FeverDisk results were characterized in reference to these molecular methods.

#### Definition of FeverDisk assay cut-off values and false positives

False positives are an important factor influencing the specificity of a diagnostic detection platform and could lead to unnecessary treatment. On the other hand, false negatives are very critical as they lead to patients not receiving treatment. In order to properly determine clinical sensitivity and specificity, one would need a large collection of samples for statistical reasons including a significant number of negatives (characterized with a reference method), which were not available in our studies in Senegal and Sudan.

Nevertheless, we attempted to define the cut-off values which can be fine-tuned in future work. Any isothermal amplification, if it runs for too long, will produce unspecific results due to primers’ tendency to self-hybridization [49] and therefore cut-offs have to be determined. In preceding work, different cut-off limits were set for the benchtop CHIKV and DENV LAMP assays [30,32]. Although we used the same primers, the FeverDisk assay is a different test with different components and conditions (dried primers, lyophilized amplification reagents, *in situ* extraction). Therefore, the cut-off had to be assessed separately for the FeverDisk. Indeed the viral RT-LAMP assays with low concentrations of primers needed longer run times and generated false positives much later, than DNA-detecting assays for *Plasmodium* and bacterial assays with higher primer concentrations and shorter run time. Each assay on the FeverDisk had a distinct presumptive cut-off value [50] and the assays were divided into three groups namely the *Plasmodium*, the bacterial, and the viral assays as described below.

Among the *Plasmodium* assays that were found positive (*P*spp and *Pf*), the *Pf* assay is the slowest based on experimental data. The highest *T*_*p*_ result for *Pf* among all tested FeverDisks was at 40 min (sample 274443-A, Table 1), which corresponded to a benchtop PCR Ct of 35.82. Such high Ct values are typical at a concentration close to the limit of detection, therefore a *T*_*p*_ of 44 min is considered as the cut-off value for all *Plasmodium* assays (a 10% increase in 40 min was considered; this 10% increase covers a 3-fold standard variation).

For the bacterial assays, given the fact that there were no bacteria-containing patient samples available at the biobanks, but only bacterial cultures, we used spiked samples in order to demonstrate the integrated assay performance and define the LOD (Table 3). Consequently we did not have FP in these samples. Therefore, for the purpose of this work, we state that the cut-off time is the assay runtime itself, i.e. 60 min, derived from the benchtop assays. This can be fine-tuned in the future when true bacteria positive samples will be tested.

For the CHIKV and DENV viral assays, the criterion is the appearance of CHIKV and DENV4 at *T*_*p*_ 64 min in sample S.16-A (disk #10, Table 1), which was a clear false positive as the sample was from an *S*. Typhi culture and therefore impossible to include any other target. This *T*_*p*_ value was therefore set as a cut-off value for all CHIKV and DENV assays. For the ZIKV LAMP, a cut-off value was not determined due to the lack of FeverDisk-positive samples that would be needed to define the FP. Nevertheless, we used, as an approximate guidance, the value of 45 min that was derived by Lopez-Jimena et al from benchtop ZIKV LAMP assays [37].

In case of suspected miscalculation of the *T*_*p*_ by the algorithm, the real-time amplification curves and/or confirmatory experiments were used in order to derive the true positive/negative results. The only two cases where this was needed were: (i) sample 274754 (Fig 4), where a late amplification curve for DENV2 co-infection appeared and would be considered negative according to the algorithm (*T*_*p*_ > 65 min). However, because it was very late, it had not had enough time to reach the plateau phase and therefore the intensity of the fluorescence was not high enough to be quantified by the algorithm that we used. Furthermore, a benchtop LAMP for DENV2 confirmed the FeverDisk result as true positive. (ii) Sample 267175 where the *T*_*p*_ for *P*spp was calculated by the algorithm to be 55 min, was considered a true negative due to the amplification curve drift which was miscalculated by the algorithm. Also, all positive viral assays below this *T*_*p*_ were confirmed true positive with reference methods.

Overall, the results from Senegal and Sudan revealed that in terms of assay speed, the malaria assays (*P*spp and *Pf*) were the most rapid in the benchtop results as well as the FeverDisks, while the DENV2 was the slowest with both methods. Thus, to encompass all cut-off values all graphs in this manuscript are set to 65 min and a summary of the false positives is given in Table 4.

**Table 4:** FeverDisk false positive results. The results are considered as such, when the *T*_*p*_ values are higher than those that were specified, i.e. *T*_*p*_ > 44 min for the *Plasmodium* assays; *T*_*p*_ > 60 min for the bacterial assays; *T*_*p*_ > 64 min for the CHIKV, DENV viral assays.

Sample	Confirmed pathogen with reference method	Other pathogens that were found false positive with the FeverDisk and their corresponding LAMP *T*_*p*_ (min)
C74-B	Malaria	DENV2 (93)
274755-A	CHIKV	*P*spp (71)
274438-B	CHIKV	DENV2 (91)
S.16-A	*S*. Typhi	CHIKV (71) DENV4 (63) *P*spp (80) *P*. *falciparum* (81) DENV2 (80)
S.pT1-A	*S*. Paratyphi A	DENV2 (95)
#14 (Sudan)	Malaria	ZIKV^a^ (62)

^a^: Primer-design approach by Phylogeny, Principal Component Analysis and LAVA

#### False negative results

Regarding the FN results (8/59 cases), some assumptions and possible explanations are expressed below.

In two FeverDisk cases, 274530 and B32, that 200 μL of the sample was not available for the disk, 50 μL molecular grade water and 50 μL PBS, respectively, were added to 150 μL of the sample, and the results were FN on the disk. However, in three other FeverDisk cases (one for 274754 and two for C71), when the same dilution had to be done, the results were TP on the disk. Therefore, the impact of the dilution factor of 3/4 sample and 1/4 water or buffer for the FN samples can be regarded as negligible.

Because we performed regular quality control evaluation of the reagents before integration into the FeverDisk, the possibility of reagent failure is unlikely. Especially in terms of RNA-lyopellet stability, preliminary accelerated long-term storage tests of the lyopellets were performed at 99% humidity and 50°C temperature for 12 weeks, which corresponds to stability of 5.6 years storage at 4°C or 1.6 years at 22°C. The tests were done with RT-lyopellets and primers targeting DENV1 virus to test cold chain independence for the transfer of the cartridges to African settings.

The above show that the most likely explanation for the false negatives was the concentration of the pathogen being below the detection limit of the method. A spiked internal control (IC) was not used in the current study but will be included in an extra chamber in the next step of the development. Positive and negative controls for all individual assays were not included nor required for closed diagnostic cartridge system like the FeverDisk, according to the RiliBÄK (Richtlinien der Bundesärztekammer) [51].

### Epidemics and diagnostic value

In cases of epidemics, caused by vector-borne diseases, the acute fever symptom overlaps with that of endemic diseases. Given the sudden and massive nature of arbovirus epidemics, there is a tendency to screen only for the epidemic-specific pathogen. Consequently, symptomatic treatment of the epidemic fever may fail to address other possible underlying disease(s) and specific treatment may not be offered at all.

In the 2014 Ebola epidemic about 90% of suspected cases matching the case definition were tested negative for Ebola virus. However, the cause of their fever was not determined due to lack of provision of testing for differentials [52]. In another study it was clearly shown that a sudden DENV epidemic in Tanzania overlapped with endemic malaria in 2014 [53]. Especially during the 2015–2016 ZIKV outbreak, there were urgent requests for combined tests to differentially diagnose DENV, CHIKV and ZIKV fevers because all these three diseases exhibit very similar clinical symptoms [54,55].

The FeverDisk demonstrated the ability to detect double infections in samples from a chikungunya epidemic in Senegal in 2015, namely co-infections of CHIKV with DENV2 (sample 274754) and CHIKV with DENV4 (sample 274755). In the case of sample 274443, (which was expected CHIKV-positive but had undergone RNA degradation between collection and testing time), a co-infection with *P*. *falciparum* malaria, which had been missed by the routine CHIKV analysis during the epidemic in 2015, was additionally detected by the FeverDisk, thus highlighting the impact that this system can have. Additionally, the LabDisk responded successfully to the spiking of three bacteria simultaneously in order to define their LOD as a “simulated” condition of co-infections. These examples clearly demonstrate that diagnostic tools with multiple parameter detection capability, like the FeverDisk, have the unique advantage to detect endemic and epidemic pathogens simultaneously and therefore can help to improve decisions for suitable treatment.

Furthermore, the FeverDisk proved to be a useful tool for virus serotyping, as shown by the DENV1 results for samples 267175 and 267197. By including the specific primer sets for DENV1-4 in four reaction chambers, one experimental run was sufficient to determine the DENV serotype. The approach to detect the DENV types individually guarantees that all of the documented variation of DENV genomes is covered [32]. Although this is in principle not necessary for patient management and therapy prescription, it is important for epidemiological and entomological studies and mapping of prevalences [56]. Similarly, for species identification of *Plasmodium*, the fact that the FeverDisk is capable of detecting multiple targets allows the inclusion of individual *Plasmodium*-specific primers, as well as the pan-malaria, *Plasmodium* species (*P*spp) assay. It was shown in this work that in total 14 FeverDisks were true positive for malaria at both test sites, and the *P*spp assays were always concordant with the *Pf* assay. Thus, the FeverDisk platform could support comprehensive data acquisition for vector-borne pathogens (*Plasmodium*, DENV, CHIKV, ZIKV) [57,58].

### Adaptability and universality of platform components

The Senegal primer panel of the FeverDisk was extended to include ZIKV [37] in the Sudan test series (Table 2) in response to the large outbreak in Latin America and Africa. A ZIKV positive sample was not determined by the FeverDisk (ZIKV negativity was not confirmed by benchtop RT-PCR or RT-LAMP) but this change in the Sudan test panel demonstrates the adaptability of the platform, keeping the cartridge design and non-specific reagents (extraction kit and reaction mix lyopellet), while changing only the primer panel. Furthermore, this adaptability was also shown when performing the LOD characterization using three simultaneously spiked bacteria, in which case the panel was modified to have four times the primers of the three bacteria (thus filling 12 reaction chambers).

The extraction reagents as well as the lyophilized amplification reagents were proven to be of universal functionality, as they were compatible with parasites and viruses (tested in Senegal and Sudan), as well as bacteria (tested during the bacterial LOD experiments in Germany).

In terms of specimens, the FeverDisk was shown to be compatible with several sample matrices, such as whole blood, serum, supernatant of infected cells, stool culture (even mixture of serum/molecular grade water, whole blood/PBS). This feature is very important for a system used as a platform aiming to be applied in a broad range of different laboratories/settings, which may have different collection methods and sample types.

### Usability assessment and feedback by end-users for future outlook

The LabDisk has been used in the past for the detection of either only bacteria or only viruses [38,59]. In other cases, only reduced versions of the disk had been tested for tropical infections, namely Rift valley fever virus [60] and Yellow fever virus [61] including either extraction or amplification units but not using pre-stored reagents. The test series in Senegal and Sudan constituted the first time a fully integrated sample-to-answer LabDisk was used in Africa with pre-stored and universal reagents suitable for bacteria, viruses, and parasites detection in single- or co-infection configuration and with the ability to handle different sample matrices. This can open the way for automated analysis of samples, without the need for manual sample handling, thereby reducing the time to result and possible handling errors. The platform is also suitable for remote settings/sentinel sites due to its low power consumption requirements (isothermal amplification instead of thermocycling) [62–64] and cold chain independence.

One objective of the demonstration experiments was to assess the training requirements and acquire feedback from the end-users regarding usability assessment. Using images and video materials, as well as hands-on execution, the training was completed within two days and included disk handling, mounting on the processing device, and the use of the device software. The users commented on the usefulness of the FeverDisk in terms of its automation and multi-target testing capability. The compatibility with several different sample matrices (serum, whole blood, supernatant of cells, bacterial cultures) was also marked as an advantage.

The recommendations from the end-users, which can be used as an outlook by the developers, concerned the user-friendliness of the software interface and the use of alternative power sources, e.g. battery or solar panel driven device, as electricity cuts affected some experimental results. Furthermore, it was strongly recommended to develop a library of FeverDisks/panels tailored to different geographic, endemic and epidemic needs. From this perspective, the exchangeability of primers, as demonstrated in the three small scale studies, is of advantage. Candidate next assays to be developed and integrated include those for tuberculosis, encephalitis, meningitis and leptospirosis, as well as for West Nile virus and Yellow fever virus. On the assay development level, there is further room for improvements. Due to the fact that the current disk design uses up the space of a whole disk for one patient sample, a next generation of FeverDisks can explore the use of mechanical sample lysis (crude lysis) followed by direct amplification. This could save space on disk, so that eventually two or three patients’ samples could be tested simultaneously on one disk. In the light of recent improvements to deriving quantitative information from isothermal amplification curves [65] it would be worth exploring the capabilities of LAMP technology in order to provide quantitative, or at least semi-quantitative information that could potentially correlate the amplification results with the pathogen load. This could assist in accurate monitoring of patients during the administration of antimicrobial treatment.

Naturally, the cost was one topic that was discussed with the end-users. Given the late-stage development of the platform there is no available information on its transfer price. However, the technology developers have identified the cost-driver mechanisms and ways to overcome them, for example by using improved lyophilization methods, or transferring the cartridge manufacturing method from thermoforming of polymer foils to injection molding. In any case, the pricing of the proposed approach should not be compared against simple LFTs because the level of information and consequently the diagnostic value that they offer is quite different. Although a thorough cost/impact and health economics analysis needs to be done, it can be easily deduced that the fully automated nature of the proposed approach offers a competitive advantage against benchtop manual approaches in terms of “hidden” personnel costs that are spent for performing multiple extractions and amplifications. Overall, as the cost is also a function of the number of targets for which the test is dedicated, it is expected that the cost per target pathogen will be more competitive than the one in currently marketed systems [20].

Next steps will include a reproducibility and production robustness analysis of the FeverDisk cartridges. Consequently, the system will be tested in a prospective study, with recruited patients ideally in settings with different endemic and epidemic profile, including several well-characterized negative samples, in order to determine the clinical sensitivity and specificity as well as the positive/negative predictive value of the platform. In these studies we will also compare the FeverDisk performance with fresh versus biobanked samples. Given the fact that the system is capable of providing multiple target results, a clinical algorithm interface that can offer decision support for the clinician is deemed necessary. Such an algorithm, in combination with the ability to distinguish viral from bacterial infections, could be an added value in management of antimicrobial resistance.

## Supporting information

S1 Text Protocols. Section A. Protocol for preparation of bacterial cultures. Section B. Protocol for manual nucleic acid extraction and purification. Section C. Protocol for FeverDisk production.
(PDF)

S1 Table Microfluidic protocol for fully automated nucleic acid extraction and amplification. (PDF)

S2 Table Total volume of primer and trehalose solutions dispensed into the FeverDisk for pre-storage in dry format. Final primer concentration for each LAMP reaction is also provided.
(PDF)

S1 Fig FeverDisk microfluidic process automation. (PDF)

S2 Fig Real-time LAMP curves from two FeverDisks testing the CHIKV sample 274443. (PDF)

S3 Fig Various single-infection pathogens detected successfully with the LabDisk. (PDF)

S4 Fig Laboratory tests on spiked bacteria samples. (PDF)
