# Supplementary material for: Fully automated point-of-care differential diagnosis of acute febrile illness
Source: PLoS Negl Trop Dis. 2021 Feb 25;15(2):e0009177. doi: 10.1371/journal.pntd.0009177 (PMC7906357; doi:10.1371/journal.pntd.0009177)
Supplement: S1 Text — Section A. Protocol for preparation of bacterial cultures. Section B. Protocol for manual nucleic acid extraction and purification. Section C. Protocol for FeverDisk production. (PDF) [file pntd.0009177.s001.pdf]

Section A, S1 Text. Protocol for preparation of bacterial cultures. For pre-enrichment, the fecal samples were cultured in 1:10 buffered peptone water (2.5 g of feces and 22.5 mL of medium) and incubated at 37 ºC for 24 h. Subsequently, 0.5 mL of the resulted suspended solution was transferred into the Rappaport-Vassiliadis *Salmonella* enrichment broth and incubated at 42 °C for 24 h. Finally, the broth culture was used to innoculate XLD agar medium (Becton Dickinson) and Hektoen enteric agar (Oxoid) plates, which were incubated at 37 ºC for 24 h. Suspected *Salmonella* colonies were confirmed by biochemical (Api 20E, ref 20100; bioMérieux, France) and serological tests (Statens Serum Institut). Serotyping was performed by slide agglutination in a Kauffmann–White scheme [1].

Section B, S1 Text. Protocol for manual nucleic acid extraction and purification. Lysis was performed by adding 150 µL lysis buffer to 200 µL sample. The total volume was mixed by pipetting 5 × up and down, then incubated for 10 min at 37 °C, while thoroughly mixing. For binding of nucleic acids to the magnetic beads, 440 µL binding buffer and 30 µL beads were added to the lysate. The mixture was thoroughly mixed by pipetting 5 × up and down and then incubated for 10 min at 37 °C on a shaker. After binding, the tube was placed in a magnet rack and beads were collected at the tube wall for 3 min. After discarding the supernatant, the tube was removed from the magnet rack and 200 µL washing solution 1 were added. Magnetic beads were re-suspended by pipetting 5 × up and down, and subsequently placed in the magnet rack for 3 min. Afterwards, the supernatant was discarded. Same steps were repeated using washing solution 2. To elute the nucleic acids, the tube was removed from the magnet rack and 200 µL elution buffer were added. Magnetic beads were re-suspended by pipetting 5 × up and down and the suspension was incubated for 10 min at 56 °C, while thoroughly mixing. If not processed immediately, eluates were stored at -20 °C.

Section C, S1 Text. Protocol for FeverDisk production. Microfluidic structures were designed using SolidWorks2013 (Dassault Systèmes SolidWorks Corp., Waltham/MA, USA). FeverDisk cartridges were produced by the Hahn-Schickard Lab-on-a-Chip Foundry [2] as described by Focke et al [3]. Using micro-milling (EVO, Kern Microtechnik GmbH, Germany), a negative master structure was milled in poly(methyl methacrylate) (PMMA). From this master, a poly(dimethylsiloxane) (PDMS) mold was cast for use in micro-thermoforming. FeverDisk cartridges for experiments in Dakar, Senegal were replicated in cyclo-olefin polymer (COP) (COP ZF 14-188, TOPAS Advanced Polymers GmbH, Germany) and for experiments in Khartoum, Sudan and in Reinfeld, Germany in cyclo-olefin copolymer (COC) (COC6013-8007, 200 µm thick, TOPAS Advanced Polymers GmbH, Germany) using a hot embossing machine (HEX01, Jenoptik AG, Germany). COC exhibits better adhesive sealing properties, and with no observed influence on the assay or biochemical components performance. To provide hydrophobic surface properties in the microfluidic structures for nucleic acid extraction, the following chambers of the cartridge were coated with a solution of 0.5 % w/w Teflon (Teflon Amorphous Flouropolymer, DuPont, India) in Fluorinert™ FC-770 (art. # F3556-100ML, Sigma-Aldrich Chemie GmbH, Germany): Binding chamber, washing 1 chamber, washing 2 chamber, elution chamber. The solvent was allowed to evaporate for 15 min. Magnetic silica beads (innuPREP MP Basic Kit A, art. # 845-KS-4900500, Analytik Jena GmbH, Germany) were pre-stored in the bead pre-storage chamber next to the binding chamber, following the steps indicated in S1 Fig: A suspension of 30 µL beads and 15 µL 250 mM D(+)trehalose dihydrate (art. # T9531, Sigma-Aldrich Chemie GmbH, Germany) dissolved in DNase/RNase-free distilled water (art. # 10977-049, Thermo Fisher Scientific, USA) was dispensed into the bead pre-storage chamber, then allowed to dry at 50 ^{o}C for 1 h.

Primer mixes were prepared as follows: All primer mixes were mixed (v/v = 1/1) with a solution of 200 mM D(+)trehalose dihydrate (art. # T9531, Sigma-Aldrich Chemie GmbH, Germany) in DNase/RNase-free distilled water (art. # 10977-049, Thermo Fisher Scientific, USA). Primer mixes for DENV2 and non-virus targets were previously heated up to 95 °C for 5 min for melting primer dimers and were cooled immediately on ice before mixing with trehalose. The primer mix volumes dispensed into the reaction chambers of the FeverDisk as well as the final primer concentrations in a LAMP reaction are listed in S2 Table.

Stick-packs for buffer pre-storage [4, 5] were produced on a customized stick-pack machine (SBL50, MERZ Verpackungsmaschinen GmbH, Germany). The sealing of the composite foil-pouches was achieved using pressure and temperature instead of ultrasonic-welding. The stick-pack sealing allowed for liquid release on demand during operation since the binding buffer should be released at rotation frequency f = 70 Hz into the lysate after completion of lysis, while all other buffers should be released right in the beginning of the FeverDisk automated process (at f = 50 Hz, see S1 Table for details). Stick-packs were placed into the corresponding chambers as indicated in the manuscript Fig.1. Lyopellets ("V6.30", custom developed by Mast Group Ltd, UK) were placed into the mixing chamber (see manuscript Fig.1). Filter membranes (art. # PTFEPET 02205, Millipore, Burlington, MA, USA) were cut to a diameter of 4 mm and were mounted onto the air vent holes in order to prevent possible amplicon or pathogen containing aerosols to leave the cartridge. Pressure-sensitive adhesive sealing foil (art. # 900 360, HJ-BIOANALYTIK GmbH, Germany) was laser cut (PLS3.6, Universal Laser Systems Inc., Austria) to pieces with an outer diameter of 130 mm, which were used for sealing the FeverDisk cartridges. For the purpose of stable storage and shipment, cartridges were packaged into petri dishes (art. # 294763910, neoLab, Germany) with one desiccant bag (art. # N078.1, Carl Roth GmbH & Co. KG, Germany) per dish providing a dry atmosphere. Each petri dish was packaged in a sealed aluminum pouch (Machine: VP440, VAMA Maschinenbau GmbH,

Germany; Bag: A20T – 14 cm x 20 cm, Long Life for Art, Germany) featuring a nitrogen atmosphere. FeverDisks packaged in aluminum pouches were stored at ambient temperatures without the need for refrigeration or cold chain during transportation.

References

1. Grimont P, Weill F-X. (2007). Antigenic Formulae of the Salmonella serovars, (9th ed.) Paris: WHO Collaborating Centre for Reference and Research on Salmonella. Institute Pasteur. 1-166.
2. https://www.hahn-schickard.de/en/production/lab-on-a-chip-foundry, Accessed November 23rd 2019.
3. Focke M, Stumpf F, Faltin B, Reith P, Bamarni D, Wadle S, et al. Microstructuring of polymer films for sensitive genotyping by real-time PCR on a centrifugal microfluidic platform. Lab Chip. 2010; 10(19):2519-2526. https://doi.org/10.1039/c004954a. PubMed PMID: 20607174.
4. van Oordt T, Barb Y, Smetana J, Zengerle R, von Stetten F. Miniature stick-packaging - an industrial technology for pre-storage and release of reagents in lab-on-a-chip systems. Lab Chip. 2013; 13(15):2888-2892. https://doi.org/10.1039/c3lc50404b. PubMed PMID: 23674222.
5. van Oordt T, Barb Y, Zengerle R, von Stetten. Lamination of polyethylene composite films by ultrasonic welding. Investigation of peel behavior and identification of optimum welding parameters. J Appl Polym Sci. 2014; 131(10):n/a. https://doi.org/10.1002/app.40291.
