# Supplementary material for: Fully automated point-of-care differential diagnosis of acute febrile illness
Source: PLoS Negl Trop Dis. 2021 Feb 25;15(2):e0009177. doi: 10.1371/journal.pntd.0009177 (PMC7906357; doi:10.1371/journal.pntd.0009177)
Supplement: S1 Table — (PDF) [file pntd.0009177.s002.pdf]

S1 Table. Microfluidic protocol for fully automated nucleic acid extraction and amplification.

Step[a]	#	Action description	Rotation frequency [Hz]	Rotation acceleration [Hz s^{-1}]	Temperature [°C]	Duration [s][b]
Sample addition	0-1	Add 200 µL sample to the sample inlet using a pipette. Seal with tape[c]	0	N/A	N/A	N/A
Lysis	1-1	Stick-pack opening I[d]	50	11		30
	1-2		10	10		0
	1-3	Set lysis temperature			37	0
	1-4	Lysis incubation loop[e]	2 s @ 10 Hz/ 2 s @ 18 Hz	10		600
Binding	2-1	Stick-pack opening II	70	11		15
	2-2	Binding step	2 s @ 10 Hz / 2 s @ 18 Hz	10		600
	2-3	Transfer beads using GTM[f]	2	5		120
Washing I	3-1	Spin down beads	40	5		10
	3-2	Mixing loop[e]	2 s @ 14 Hz/ 2 s @ 20 Hz	10		6
	3-3	Sediment beads	40	10		0
	3-4	Sediment beads	15	10		0
	3-5	Transfer beads using GTM[f]	1.5	10		60
	3-6	Sediment beads	40	5		0
Washing II	4-1	Repeat bead transfer protocol 6×: steps 3-1 to 3-6				
Elution	5-1	Rotation frequency reduction	12	10		0
	5-2	Heating up elution buffer			56	
	5-3	Elution loop[e]	2 s @ 12 Hz/ 2 s @ 20 Hz	10		600
	5-4	Remove beads from eluate	2	1		10

1st TCR[g] actuated valving [1]	6-1	Cooling for valve actuation	3		45	0
Centrifugo-pneumatic inward pumping [2]	7-1	1st compression step	60	5		10
	7-2	1st inward pumping step	5	11		10
	7-3	2nd compression step	70	5		10
	7-4	2nd pumping step	5	11		10
TCR actuated mixing [3]	8-1	Remove residual liquid from channels	20	10		5
	8-2	Rotation frequency reduction for bubble mixing	6	10		0
	8-3	Heating for bubble mixing			60	
	8-4	Loop[e]: Combined shake-mode and bubble mixing	1 s @ 6 Hz/ 2 s @ 3 Hz	10		20
	8-5	Cool down for restoring initial pressure conditions	30	5	45	20
	8-6	Remove residual liquid from channels	20	10		5
	8-7	Loop: Repeat 4× steps 8-2 to 8-6				
2nd TCR actuated valving [1]	9-1	Heat up to prepare valving			60	30
	9-2	Remove residual liquid from channels	50	10		0
	9-3	Start cooling for valving			45	
	9-4	Set rotation frequency for valving	8	5		10
Metering and cent.-pneum. Aliquoting [4]	10-1	Metering	11	5		20
	10-2	Centrifugo-pneumatic aliquoting	50	3		0

LAMP reaction & detection	11-1	Set rotation frequency	5	5		
	11-2	Set LAMP reaction temperature			64	30
	11-3	Sequential (chamber 1 → chamber 12) detection in FAM channel				
	11-4	LAMP reaction loop: Repeat steps 11-1 to 11-3 for 2 h				

[a]: If no value is stated for a parameter, it remains constant, as stated before.

[b]: "Duration" refers to the time a set of parameters is kept constant, before the device calls a different parameter. The time starts, when the given parameters (frequency, acceleration, temperature, etc.) are reached.

[c]: Art. # 900 360, HJ-BIOANALYTIK GmbH, Germany.

[d]: At the frequency of 50 Hz open simultaneously the stick-packs containing the lysis buffer, the washing solution I, the washing solution II and the elution buffer.

[e]: During loops, the described operations are repeated until the time given is over.

[f]: Gas-phase transition magnetophoresis [5].

[g]: Temperature change rate.

N/A: Parameter not applicable and/or not controlled by the device.

References

1. Keller M, Czilwik G, Schott J, Schwarz I, Dormanns K, von Stetten F, et al. Robust temperature change rate actuated valving and switching for highly integrated centrifugal microfluidics. Lab Chip. 2017; 17(5):864-875. https://doi.org/10.1039/c6lc01536k. PubMed PMID: 28181607.
2. Zehnle S, Rombach M, Zengerle R, von Stetten F, Paust N. Network simulation-based optimization of centrifugo-pneumatic blood plasma separation. Biomicrofluidics. 2017; 11(2):24114. https://doi.org/10.1063/1.4979044. PubMed PMID: 28798850; PubMed Central PMCID: PMC5533477.
3. Hin S, Paust N, Keller M, Rombach M, Strohmeier O, Zengerle R, et al. Temperature change rate actuated bubble mixing for homogeneous rehydration of dry pre-stored reagents in centrifugal microfluidics. Lab Chip. 2018; 18(2):362-370. https://doi.org/10.1039/c7lc01249g. PubMed PMID: 29297912.
4. Mark D, Weber P, Lutz S, Focke M, Zengerle R, von Stetten F. Aliquoting on the centrifugal microfluidic platform based on centrifugo-pneumatic valves. Microfluid Nanofluid. 2011; 10(6):1279-1288. https://doi.org/10.1007/s10404-010-0759-0.
5. Strohmeier O, Emperle A, Roth G, Mark D, Zengerle R, von Stetten F. Centrifugal gas-phase transition magnetophoresis (GTM) - a generic method for automation of magnetic bead based assays on the centrifugal microfluidic platform and application to DNA purification. Lab Chip. 2013; 13(1):146-155. https://doi.org/10.1039/c2lc40866j. PubMed PMID: 23142800.
