# Supplementary material for: Fully automated point-of-care differential diagnosis of acute febrile illness
Source: PLoS Negl Trop Dis. 2021 Feb 25;15(2):e0009177. doi: 10.1371/journal.pntd.0009177 (PMC7906357; doi:10.1371/journal.pntd.0009177)
Supplement: S2 Table — Final primer concentration for each LAMP reaction is also provided. (PDF) [file pntd.0009177.s003.pdf]

S2 Table. Total volume of primer and trehalose solutions dispensed into the FeverDisk for pre-storage in dry format. Final primer concentration for each LAMP reaction is also provided.

**Primer mix target**	**Total volume of primer and trehalose solutions (µL)**	**Final primer concentration for each assay**
P. spp	2.0	For all:<br>0.2 µM each of F3 and B3;<br>1.6 µM each of FIP and BIP;<br>0.8 µM each of LF and LB
P. falciparum	2.0	
P. vivax	2.0	
P. malariae	2.0	
S. Typhi	2.0	
S. Paratyphi (A)	2.0	
S. pneumoniae	2.0	
CHIKV	3.2	0.1 µM each of F3 and B3;<br>0.8 µM each of FIP and BIP;<br>0.4 µM each of LF and LB (per primer set) [1]
DENV1	4.8	For all:<br>50 nM each of F3 and B3;<br>400 nM each of FIP and BIP;<br>200 nM each of LF and LB (per primer set) [2]
DENV2	4.2	
DENV3	4.4	
DENV4	1.6	
ZIKV[a]	3.2	0.1 µM each of F3 and B3;<br>0.8 µM each of FIP and BIP;<br>0.4 µM each of LF and LB (per primer set) [3]

[a]: Same concentration for both assays, designed by the University of Stirling (Phylogeny, Principal Component Analysis and LAVA) and Mast Diagnostica GmbH (Primer Explorer V4)

References

1. Lopez-Jimena B, Wehner S, Harold G, Bakheit M, Frischmann S, Bekaert M, et al. Development of a single-tube one-step RT-LAMP assay to detect the Chikungunya

virus genome. PLoS Negl Trop Dis. 2018; 12(5):e0006448. https://doi.org/10.1371/journal.pntd.0006448. PubMed PMID: 29813065; PubMed Central PMCID: PMC5973553.

2. Lopez-Jimena B, Bekaert M, Bakheit M, Frischmann S, Patel P, Simon-Loriere E, et al. Development and validation of four one-step real-time RT-LAMP assays for specific detection of each dengue virus serotype. PLoS Negl Trop Dis. 2018; 12(5):e0006381. https://doi.org/10.1371/journal.pntd.0006381. PubMed PMID: 29813062; PubMed Central PMCID: PMC5973574.

3. Lopez-Jimena B, Bakheit M, Bekaert M, Harold G, Frischmann S, Fall C, et al. Development and Validation of Real-Time RT-LAMP Assays for the Specific Detection of Zika Virus. In: Kobinger G, Racine T, editors. Zika Virus. Methods in Molecular Biology. Humana, New York, NY. 2020. pp. 147-164.
