# Supplementary material for: Fully automated point-of-care differential diagnosis of acute febrile illness
Source: PLoS Negl Trop Dis. 2021 Feb 25;15(2):e0009177. doi: 10.1371/journal.pntd.0009177 (PMC7906357; doi:10.1371/journal.pntd.0009177)
Supplement: S1 Fig — (PDF) [file pntd.0009177.s004.pdf]

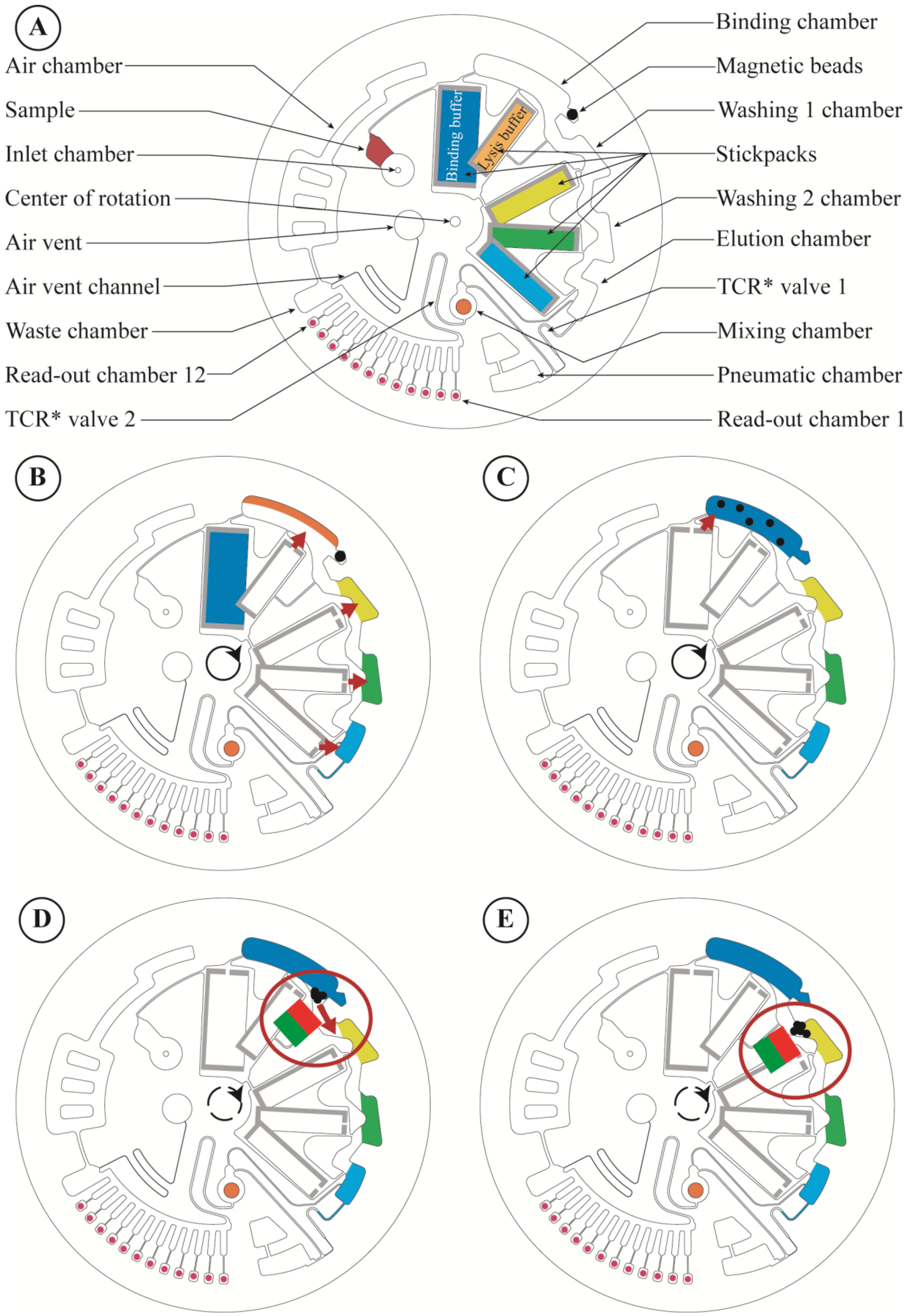
A
Air chamber
Sample
Inlet chamber
Center of rotation
Air vent
Air vent channel
Waste chamber
Read-out chamber 12
TCR* valve 2
Binding chamber
Magnetic beads
Washing 1 chamber
Stickpacks
Washing 2 chamber
Elution chamber
TCR* valve 1
Mixing chamber
Pneumatic chamber
Read-out chamber 1
Binding buffer
Lysis buffer
B
C
D
E

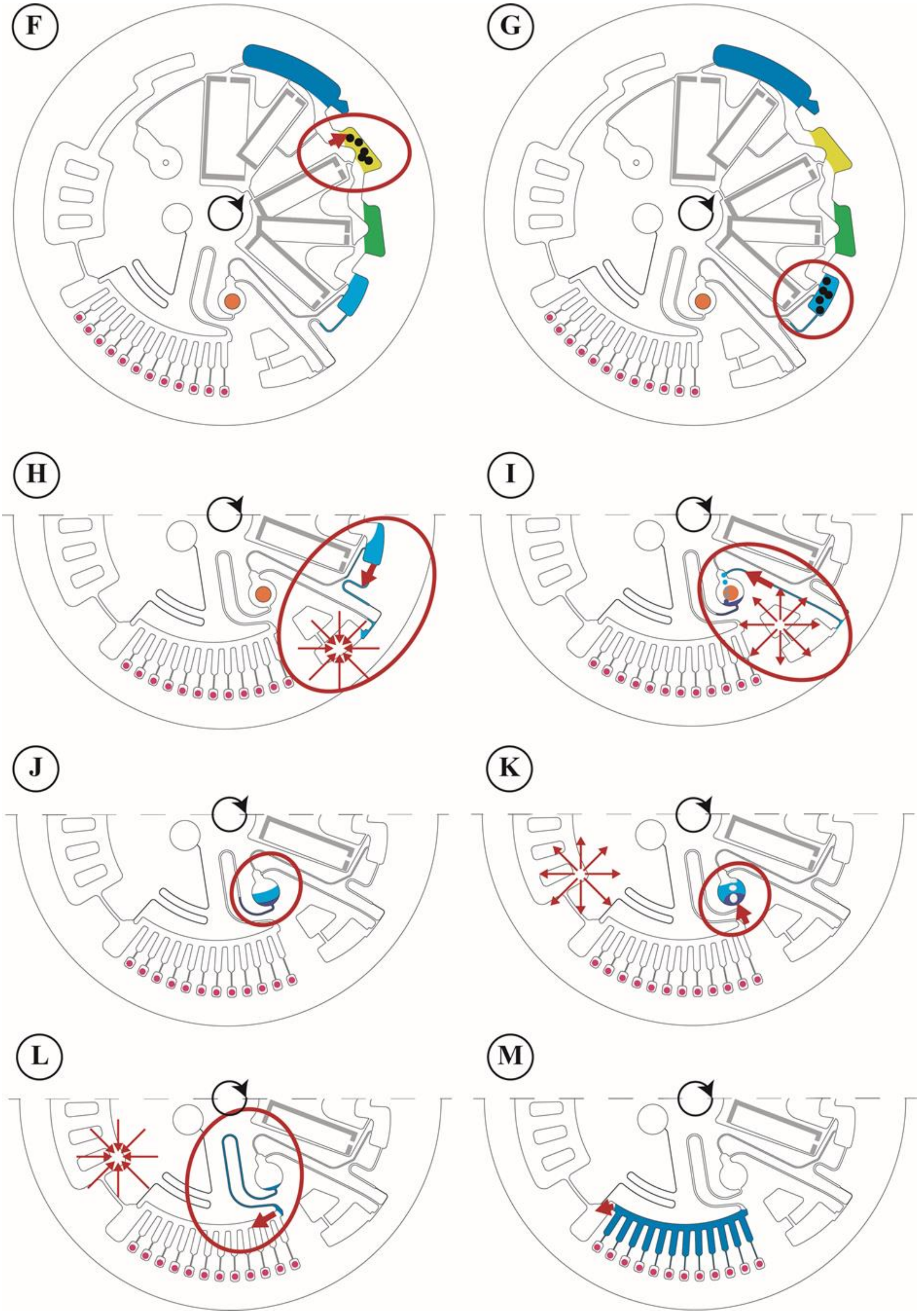
F
G
H
I
J
K
L
M

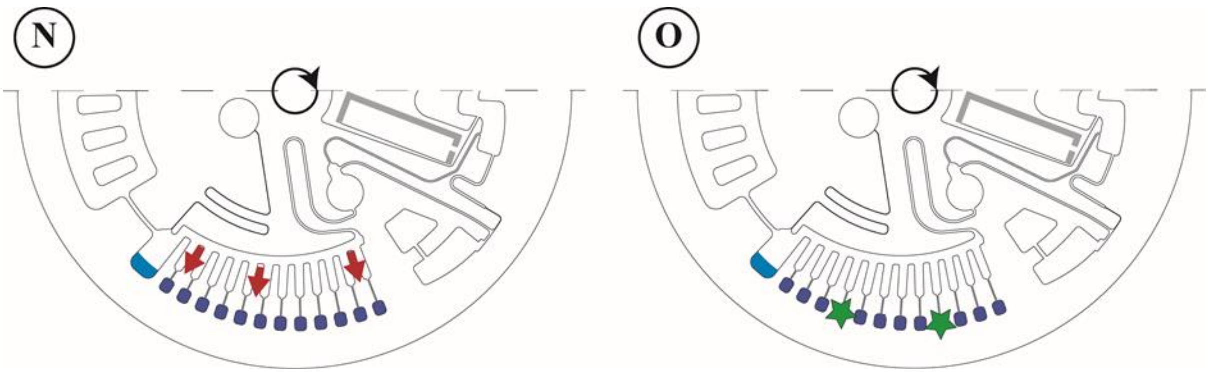

S1 Fig. FeverDisk microfluidic process automation. (*) TCR: Temperature change rate. All drawings shown are in top-view perspective. (A) Overview on the FeverDisk cartridge, which features an outer diameter of 130 mm. In the stick-packs, binding buffer (BB), lysis buffer (LB), washing buffer 1 (WB1), washing buffer 2 (WB2) and elution buffer (EB) are pre-stored, respectively. Each buffer is positioned radially inwards from the microfluidic chamber, in which it is required during the nucleic acid extraction process (e.g. the elution buffer stick-pack is located radially inwards from the elution chamber). Magnetic beads are pre-stored in dry form in a small reservoir next to the binding chamber. Lyophilized LAMP reaction mix is pre-stored in the mixing chamber and dry primer mixes for LAMP are pre-stored in the read-out chambers. The sample can be introduced into the FeverDisk using a pipette. The sample inlet is sealed after sample addition with adhesive tape avoiding contamination of the ambient with potentially hazardous biological or chemical material. (B) After starting the process, the FeverDisk rotates at 50 Hz (see S1 Table, step 1-1). Lysis buffer, washing buffers and elution buffer are released from the stick-packs at this frequency. The sample is transferred into the binding chamber, and mixed therein with the lysis buffer (see S1 Table, steps 1-2 to 1-4). The low liquid level in the binding chamber prevents the magnetic beads from rehydration, because they are not in contact with the lysate. (C) After lysis, the FeverDisk rotates at 70 Hz, resulting in a release of the binding buffer from its stick-pack into the binding chamber (see S1 Table, step 2-1). The increased liquid level combined with shake mode mixing [1] allows for rehydration of the magnetic beads and

mixing of the beads with the binding buffer/lysate solution to enable nucleic acid/bead conjugation (see S1 Table, step 2-2). (D) Following binding, the FeverDisk is positioned under an external magnet, such that the magnetic beads are moved out of the binding buffer/lysate solution. The magnet is indicated by the red/green rectangle (see S1 Table, step 2-3). (E) By rotating the FeverDisk, the magnetic beads can be moved *via* a connection channel to a position radially inwards from the washing chamber 1 (see S1 Table, step 2-3). (F) Increasing the rotation frequency over a critical frequency results in release of the magnetic beads into the washing chamber 1 (see S1 Table, step 3-1), where impurities are removed from the nucleic acids by shake mode mixing (see S1 Table, steps 3-2 to 3-4). This process is repeated in total 6 times for transport of the magnetic beads from washing chamber 1 to washing chamber 2 and for transport from washing chamber 2 to elution chamber (steps not shown, see S1 Table, step 4-1). (G) Elution of the purified nucleic acids from the magnetic beads is performed for 10 min at 56 °C, applying shake mode mixing (see S1 Table, steps 5-1 to 5-4). (H) Rapid cooling results in actuation of the TCR actuated valve 1 [2] (see S1 Table, step 6-1). (I) To transfer the eluate in radial inward direction to the lyophilized reaction mix, centrifugo-pneumatic pumping [3] is applied (see S1 Table, steps 7-1 to 7-4). (J) The eluate instantly rehydrates the lyophilized reaction mix; however, the obtained solution is not yet sufficiently mixed. (K) Rigorous mixing is achieved by employing TCR actuated bubble mixing [4] (see S1 Table, steps 8-1 to 8-7). (L) To route the obtained reaction mix to the read-out chambers, the second TCR actuated valve is activated (see S1 Table, steps 9-1 to 9-4). (M) Twelve aliquots are metered according to the method of Mark et al [5] (see S1 Table, step 10-1). (N) Aliquoting of the reaction mix into the read-out chambers, wherein dry primer mixes are rehydrated. (O) Real-time LAMP reaction with signal increase in the FAM channel, in case of a positive result (see S1 Table, steps 11-1 to 11-4).

References

1. Grumann M, Geipel A, Riegger L, Zengerle R, Ducrée J. Batch-mode mixing on centrifugal microfluidic platforms. Lab Chip. 2005; 5:560-565. https://doi.org/10.1039/b418253g. PubMed PMID: 15856095.
2. Keller M, Czilwik G, Schott J, Schwarz I, Dormanns K, von Stetten F, et al. Robust temperature change rate actuated valving and switching for highly integrated centrifugal microfluidics. Lab Chip. 2017; 17(5):864-875. https://doi.org/10.1039/c6lc01536k. PubMed PMID: 28181607.
3. Zehnle S, Rombach M, Zengerle R, von Stetten F, Paust N. Network simulation-based optimization of centrifugo-pneumatic blood plasma separation. Biomicrofluidics. 2017; 11(2):24114. https://doi.org/10.1063/1.4979044. PubMed PMID: 28798850; PubMed PMCID: PMC5533477.
4. Hin S, Paust N, Keller M, Rombach M, Strohmeier O, Zengerle R, et al. Temperature change rate actuated bubble mixing for homogeneous rehydration of dry pre-stored reagents in centrifugal microfluidics. Lab Chip. 2018; 18(2):362-370. https://doi.org/10.1039/c7lc01249g. PubMed PMID: 29297912.
5. Mark D, Weber P, Lutz S, Focke M, Zengerle R, von Stetten F. Aliquoting on the centrifugal microfluidic platform based on centrifugo-pneumatic valves. Microfluid Nanofluid. 2011; 10(6):1279-1288. https://doi.org/10.1007/s10404-010-0759-0.
