# Supplementary material for: Fully automated point-of-care differential diagnosis of acute febrile illness
Source: PLoS Negl Trop Dis. 2021 Feb 25;15(2):e0009177. doi: 10.1371/journal.pntd.0009177 (PMC7906357; doi:10.1371/journal.pntd.0009177)
Supplement: S2 Fig — (PDF) [file pntd.0009177.s005.pdf]

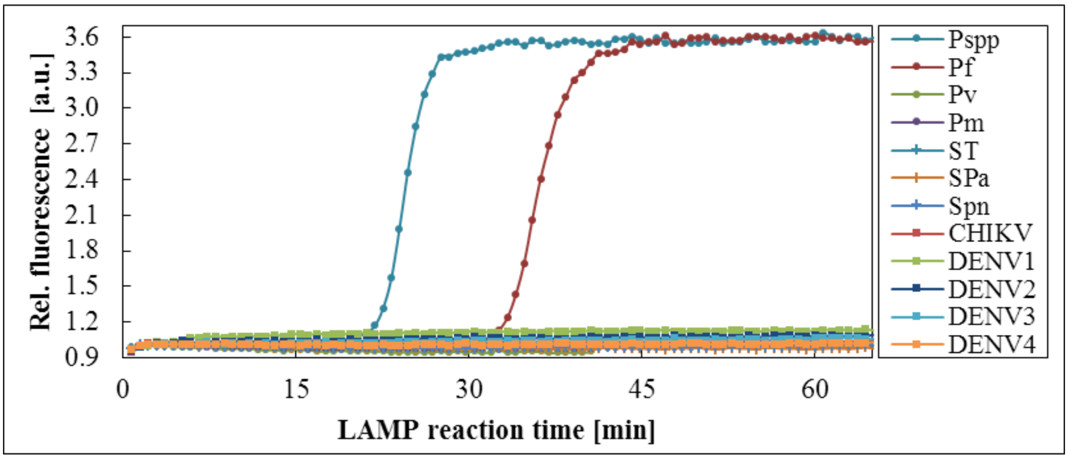

(a)

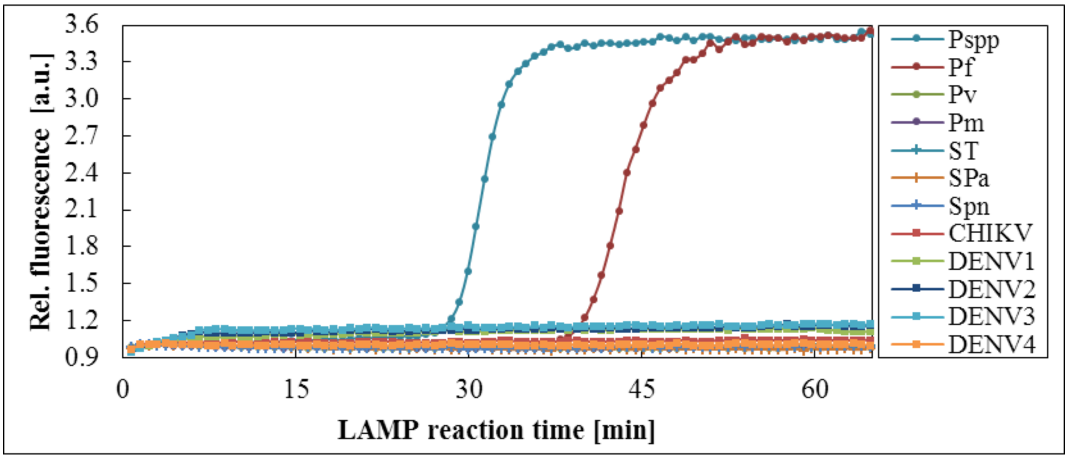

(b)

S2 Fig. Real-time LAMP curves from two FeverDisks testing the CHIKV sample 274443. The two FeverDisks results (a), (b) confirm the benchtop CHIKV-negative RT-PCR results indicating a probably RNA degradation between sample collection and FeverDisk test. Furthermore, both used FeverDisks detect an additional malaria co-infection (*P*spp and *Pf* assays), which was confirmed by benchtop PCR.
