# Supplementary material for: Fully automated point-of-care differential diagnosis of acute febrile illness
Source: PLoS Negl Trop Dis. 2021 Feb 25;15(2):e0009177. doi: 10.1371/journal.pntd.0009177 (PMC7906357; doi:10.1371/journal.pntd.0009177)
Supplement: S3 Fig — (PDF) [file pntd.0009177.s006.pdf]

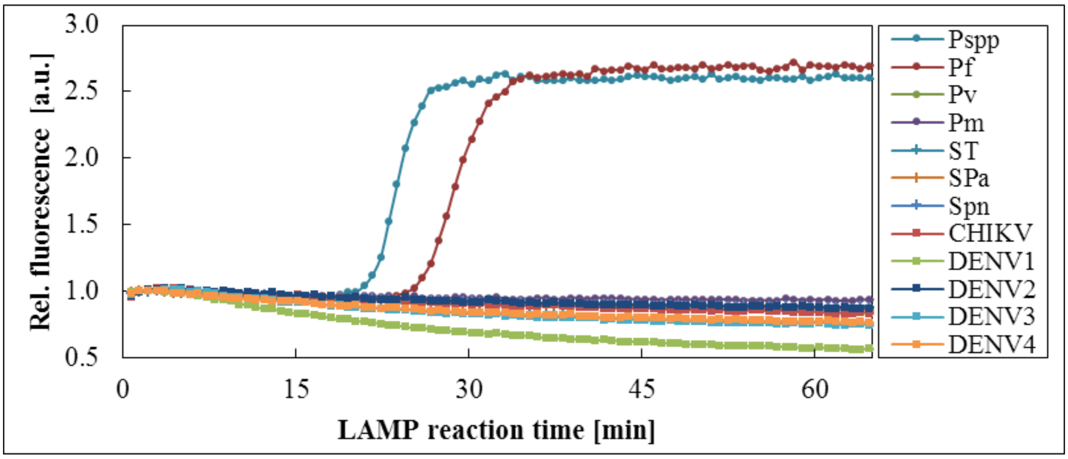

Rel. fluorescence [a.u.]
LAMP reaction time [min]
Pspp
Pf
Pv
Pm
ST
SPa
Spn
CHIKV
DENV1
DENV2
DENV3
DENV4

(a)

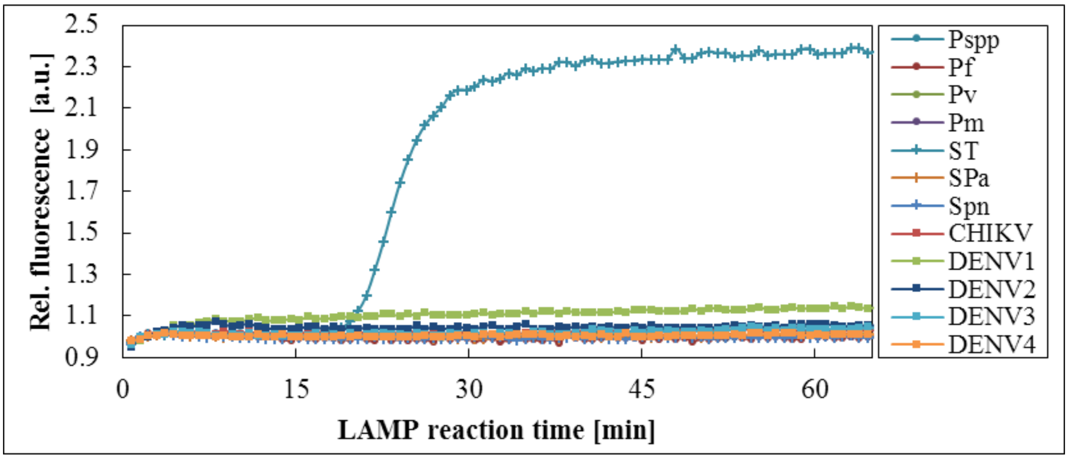

Rel. fluorescence [a.u.]
LAMP reaction time [min]
Pspp
Pf
Pv
Pm
ST
SPa
Spn
CHIKV
DENV1
DENV2
DENV3
DENV4

(b)

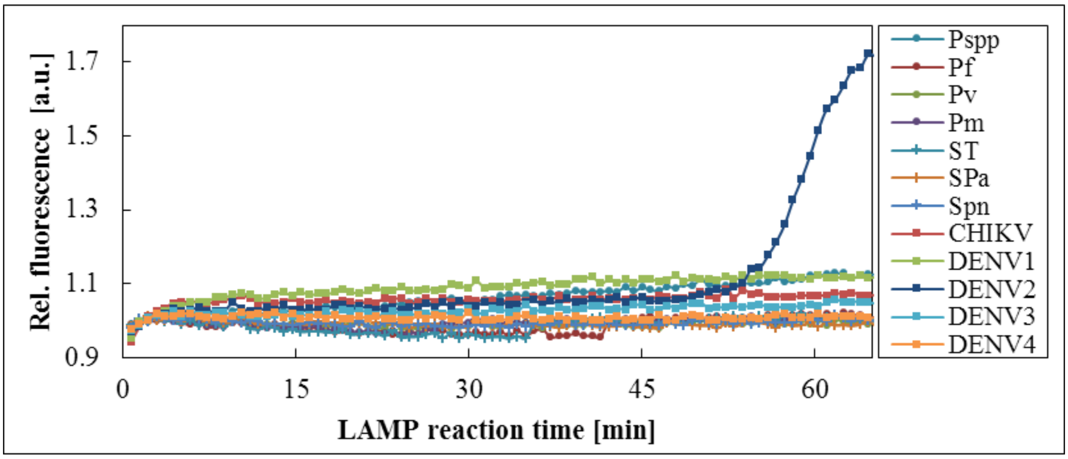

Rel. fluorescence [a.u.]
LAMP reaction time [min]
Pspp
Pf
Pv
Pm
ST
SPa
Spn
CHIKV
DENV1
DENV2
DENV3
DENV4

(c)

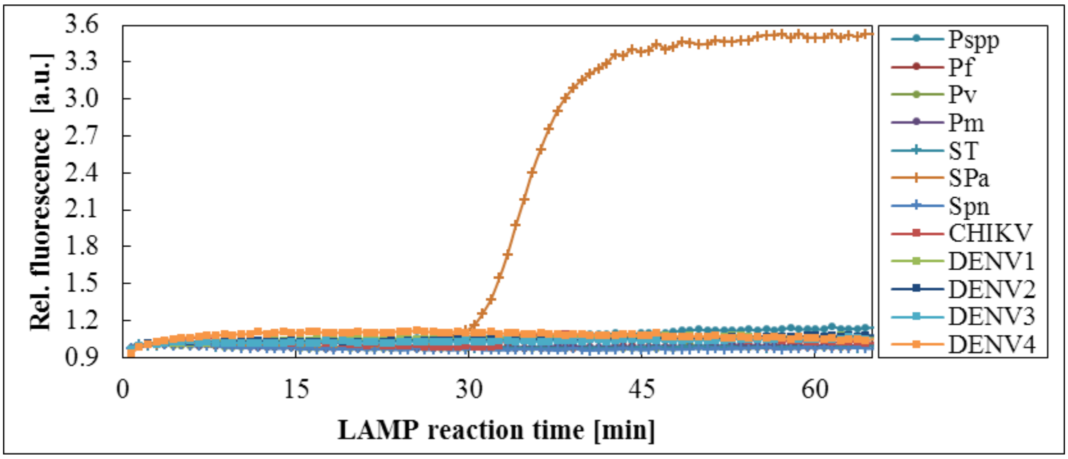

(d)

S3 Fig. Various single-infection pathogens detected successfully with the LabDisk. (a) *P*spp and *Pf* assays confirmed in sample C71. (b) *S.* Typhi assay confirmed in sample S.12. (c) DENV2 assay confirmed in sample 267150. (d) *S.* Paratyphi A assay conformed in sample S.pT1.
