# Supplementary material for: Fully automated point-of-care differential diagnosis of acute febrile illness
Source: PLoS Negl Trop Dis. 2021 Feb 25;15(2):e0009177. doi: 10.1371/journal.pntd.0009177 (PMC7906357; doi:10.1371/journal.pntd.0009177)
Supplement: S4 Fig — (PDF) [file pntd.0009177.s007.pdf]

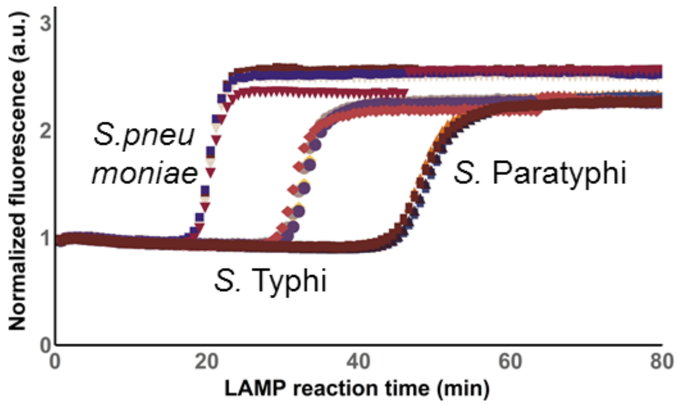

S4 Fig. Laboratory tests on spiked bacteria samples. Demonstration of three bacteria that were simultaneously spiked in blood samples, and successfully detected. This procedure was done in order to define the LOD of the FeverDisk for these bacteria. The primers for each of the three bacteria were pre-stored in 4 reaction chambers, thereby we acquired 4 amplification curves for each bacterium. For the experiment of this figure, the final concentration of each bacteria type in blood was equal to 2 x 10^4 CFU/mL.
